# Bacterial Diversity and Functional Dynamics in the Soybean ( *Glycine max* L.) Rhizosphere Under Different Organic Fertilisation

**DOI:** 10.1111/1758-2229.70370

**Published:** 2026-05-31

**Authors:** Ijeoma Emelda Osuji, Akinlolu Olalekan Akanmu, Olubukola Oluranti Babalola

**Affiliations:** ^1^ Food Security and Safety Focus Area, Faculty of Natural and Agricultural Sciences North‐West University Mmabatho South Africa; ^2^ Department of Life Sciences Imperial College London Ascot Berkshire UK

**Keywords:** 16S rRNA, bacterial diversity, cattle dung, functional redundancy, organic fertilisation, poultry manure, soil health

## Abstract

Soybean ( *Glycine max* L.) is a globally important legume for oil and protein production, yet its responses to specific organic fertilisation practices remain insufficiently understood. Organic amendments such as cattle dung and poultry manure are sustainable alternatives to inorganic fertilisers, but their effects on the soybean rhizosphere microbiome remain poorly characterised. This study investigated microbial community structure and functional diversity under poultry manure and cattle dung treatments. Rhizospheric soils were collected from treated plots, untreated controls and bulk soil and then subjected to physicochemical analysis. DNA extracted from samples was analysed using 16S rRNA gene sequencing on the Illumina NovaSeq 6000 platform, with data processed in QIIME 2 (v2019.1). Poultry manure increased available phosphorus (28%) and calcium (19%), while cattle dung enhanced potassium (22%) and magnesium (17%). Microbial community composition shifted significantly, with poultry manure promoting copiotrophic taxa such as *Burkholderia* and *Cupriavidus* and cattle dung enriching decomposers including *Paenibacillus* and *Treponema*. Alpha diversity was highest in poultry manure (Shannon index 6.2) and bulk soil (6.0) and lowest in cattle dung (5.1). Functional predictions indicated retention of core metabolic pathways, suggesting functional redundancy. Overall, organic fertilisation reshapes microbial communities while maintaining essential functions, supporting sustainable soybean cultivation.

## Graphical abstract

Soybean ( *Glycine max* L.) rhizosphere responses to organic fertilisation remain unknown. This study used 16S rRNA sequencing to assess microbial diversity under cattle dung and poultry manure. Results showed distinct community shifts, improved soil nutrients and stable core metabolic functions, highlighting organic inputs as sustainable strategies for soybean cultivation.

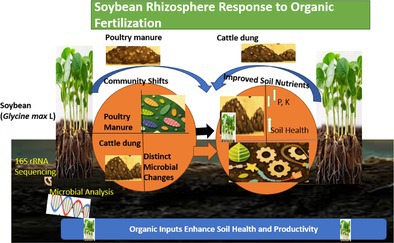

## 1 Introduction

Soybean ( *Glycine max* L.) is one of the most important legume crops across the world due to its economic, nutritional and environmental significance (Ajiboye et al. 2022). It is a plant‐based protein with higher oil content than most legumes; it is highly valued in both human and animal nutrition (Thakur et al. 2024). It is also utilised for non‐food applications such as the production of biodiesel (Modgil et al. 2021). Soybeans are cultivated in about 6% of global arable land and up to 50% of the legume growing areas are used for soybean cultivation (Semba et al. 2021). Three of the top‐producing soybean countries in the world are Argentina, Brazil and the United States of America (Klein and Luna 2021). Soybean is also one of the most commonly cultivated legumes in Africa, especially in Sub‐Saharan Africa (SSA) (Ajiboye et al. 2022). South Africa has been reported as the largest producer of soybeans in Africa, followed by Nigeria and Zambia (Sedibe et al. 2023).

The plant‐microbe interaction of legume rhizosphere has been commonly studied because of its value in agricultural production and the capacity of legumes to develop symbiotic relationships with soil rhizobia (Sohn et al. 2021). To gain insight into such interactions, there is a need to understand the dynamics of the roles and diversity of native microbial communities associated with the roots and rhizosphere (Sohn et al. 2021). The rhizosphere is the region of soil close to plant roots that contains a large number of diverse organisms (Nwachukwu et al. 2023). In addition to the high diversity of bacteria in the rhizosphere, there are interactions between plants and microbiomes in the area around the roots of soybean plants (Ayilara et al. 2023). The variation and abundance of microbial communities in the rhizosphere greatly improve the capacity of the ecosystem to cope with both abiotic and non‐abiotic stresses (Omotayo et al. 2021). The rhizosphere microbial food web is largely sustained by plant‐derived nutrients, particularly root exudates. These compounds play a crucial role in regulating microbial activity and diversity (Agarwal et al. 2024). The source and amount of exudates released from the roots, the variety of the plant, the architecture of the roots and each stage of growth are some of the factors affecting the community structure of the rhizosphere (Gu et al. 2022). Despite the complex dynamics that govern rhizosphere microbial communities, the fundamental importance and objectives of cropping systems remain inadequately understood (Hartmann and Six 2023).

Soybean cultivation faces challenges from organisms like bacterial blight ( *Pseudomonas syringae* pv. *glycinea*), bacterial pustule ( *Xanthomonas axonopodis* pv. glycines), bacterial wilt ( *Curtobacterium flaccumfaciens* pv. *flaccumfaciens*), soybean brown spot (*Septoria glycines*) and halo blight ( *Pseudomonas syringae* pv. *phaseolicola*), leading to significant losses (Dubey et al. 2021). With the recent discouragement in the use of chemical‐based fertilisers and pesticides in crop production, owing to their adverse impact on the environment (Akanmu et al. 2023). The practice of organic farming has been considered a natural approach to soil rejuvenation and crop productivity (Gamage et al. 2023). Several benefits have been accrued from the use of organic amendments sourced from cattle dung and poultry litter wastes and this involves the improvement of soil structure by stimulating microbial activity, which in turn enhances soil aggregation, increases water retention and promotes long‐term fertility (Rastogi et al. 2023). Organic fertilisation encourages a more balanced ecosystem, supports biodiversity, improves soil nutrient cycling and reduces the adverse environmental impacts of synthetic inputs (Alori et al. 2024; Gamage et al. 2023).

Due to the importance of rhizosphere bacterial communities for plant growth and performance, various investigations have been carried out using both culture‐dependent and culture‐independent approaches (Fadiji et al. 2021). Traditional microbial analysis relied on culturing methods, but over 95% of soil microbial species are non‐culturable (Giagnoni et al. 2018). However, the advancements in Next‐Generation Sequencing are revolutionising our understanding of soil microbial diversity, interactions and functions (Mishra et al. 2023). The progress in metagenomics research has facilitated the use of 16S rRNA amplicon sequencing to analyse environmental DNA, thereby offering a more comprehensive understanding of the diversity of the soil microbiome and its functional roles within the ecosystems (Mishra et al. 2023). Despite these advances, the dynamics of microbes associated with the soybean rhizosphere under different organic conditions remain largely unexplored. While plant growth responses are critical endpoints, this study focuses specifically on the rhizosphere microbiome as an early and sensitive indicator of soil biological responses to organic fertilisation. This research, therefore, investigates the microbiome community structure and the functional diversity of soybean rhizosphere soil in the fields treated with different organic fertilisation methods, namely cattle dung and poultry manure. This study particularly addresses the following questions: (i) how do different organic amendments shape rhizosphere bacterial diversity? and (ii) do these compositional changes translate into functional shifts relevant to soil fertility?

## 2 Materials and Methods

### 2.1 Experimental Layout and Sample Collection

The study was conducted at the North‐West University farm in Molelwane, Mafikeng, South Africa (25°48′11.577″S, 25°35′53.762″E), with an average rainfall of 540 mm per annum. The field experiment was conducted using a randomised complete block design (RCBD) consisting of four treatments: (i) poultry manure‐amended soil, (ii) cattle dung‐amended soil, (iii) unamended soybean rhizosphere soil (control) and (iv) bulk soil (non‐rhizosphere soil) with three replications, with each plot measuring 3 m × 3 m (9 m^2^). A spacing of 0.5 m was maintained between adjacent plots, while blocks were separated by 1 m to minimise treatment interference. Organic amendments were applied at a rate of 20 t ha^−1^ (equivalent to 2 kg m^−2^) prior to planting. Soybean seeds (cultivar PAN 1555R) were sown under standard agronomic practices.

Rhizosphere soil samples were collected at the flowering stage (7th week after planting), representing the peak of root‐microbe interaction. Rhizosphere soil samples tightly bound to the root surface were collected from each of the treatments and transported in cooler boxes containing ice to the laboratory. The soil samples were divided into two parts; the first part was stored at −20°C until soil DNA extractions were completed and the second part was stored at 4°C for the physicochemical analysis.

### 2.2 Physicochemical Analysis of the Soil Samples

The samples were sieved using a 2 mm sieve and used for physicochemical analysis. Parameters such as pH, electrical conductivity (EC), organic carbon, total nitrogen, available phosphorus and exchangeable cations (e.g., Ca^2+^, Mg^2+^, K^+^, Na^+^) were measured in the soil. pH and EC were determined using a digital metre in a soil–water suspension, organic carbon by the Walkley‐Black method, total nitrogen via Kjeldahl digestion and available phosphorus using the Bray method (Bray and Kurtz 1945). Cation exchange capacity (CEC) is estimated using ammonium acetate (Antonangelo et al. 2024).

### 2.3 DNA Extraction and Sequencing

Total genomic DNA was extracted from the soil samples using the NucleoSpin Soil Mini kit for soil DNA isolation by adhering to the manufacturer's instructions. Nanodrop Spectrophotometer (Thermo Fischer Scientific, CA, USA) was used to determine the DNA concentration. 16S rRNA gene amplicon sequencing of the DNA samples was carried out at the Novogene Laboratory, Singapore.

### 2.4 Sequencing and Data Processing

Bacterial 16S rRNA gene (V4–V5 region) libraries were prepared. PCR amplification of targeted regions was performed using the universal primers 515F (5′‐GTGCCAGCMGCCGCGGTAA‐3′) and 907R (5′‐CCGTCAATTCCTTTGAGTTT‐3′), with sample‐specific barcodes incorporated for multiplex sequencing. Furthermore, gel electrophoresis was performed to confirm the proper amplification and absence of unintended PCR products. The library was then checked with Qubit and real‐time PCR for quantification, while a bioanalyzer was used for size distribution detection. Quantified libraries were pooled and sequenced on Illumina platforms according to the effective library concentration and the amount of data required (Al‐Salameen et al. 2023; Bokulich and Mills 2013; Caporaso et al. 2012).

**FIGURE 1:**
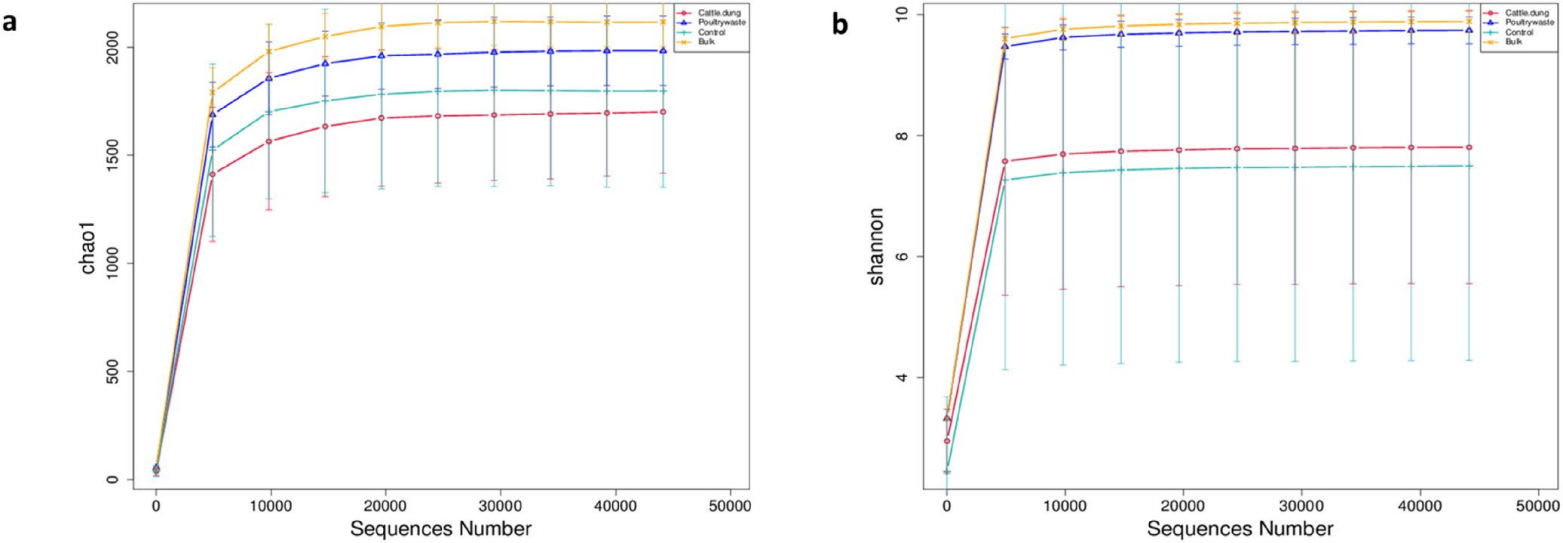
Rarefaction curves showing sequencing depth across the treatments in the (a) Chao1 and (b) Shannon indices.

The rarefaction curves based on Chao1 richness (a) and Shannon diversity (b) illustrate the sequencing depth and diversity of the bacterial community across treatments. In both cases, the curves approach saturation, indicating that the sequencing effort would be sufficient to capture the majority of microbial diversity within the respective samples. Plateauing of curves at higher sequence numbers affirms adequate sequencing coverage and reliability of diversity estimates, which is also supported with high Good's coverage values (~0.998) (Figure 1).

### 2.5 Bioinformatic Analysis and Statistics

Microbiome sequencing data were analysed using the Quantitative Insights into Microbial Ecology 2 (QIIME 2, version 2021) (Bokulich et al. 2018; Bolyen et al. 2019). Initially, raw sequencing reads were subsampled to a uniform depth of 150,000 reads per sample using SeqTK2. Demultiplexed sequences and associated metadata were imported into QIIME 2 via q2‐import. Imported sequences underwent quality filtering, trimming of the first five nucleotide reads and reads below the quality thresholds, facilitated by the q2‐demux plugin. Following quality filtering, sequence denoising was performed using the DADA2 algorithm via q2‐dada2 (Callahan et al. 2021). Amplicon sequence variants (ASVs) were identified, aligned using mafft (via q2‐alignment) and phylogenetic trees constructed utilising FastTree (via q2‐phylogeny). Taxonomic assignment of bacterial ASVs employed a pre‐trained Naïve Bayes classifier trained on the SILVA 138.1 reference database.

Then analysed abundance, Alpha diversity was conducted to assess within‐sample species richness and evenness, while the computed Beta diversity metrics, including Bray–Curtis dissimilarity and weighted/unweighted UniFrac distances, evaluated the variations in the composition of microbial community between the samples. The principal coordinate analysis (PCoA) plots, Venn diagram and Flower graph were generated to visualise the clustering patterns among different treatment groups. The sequences were deposited in the Read Archive (SRA) with Bioproject number PRJNA1177761.

Functional Prediction of the Bacterial Community: Functional profiling was performed with PICRUSt2 (version 2.3.0), referencing KEGG and MetaCyc databases. LEfSe (LDA Effect Size) was used to identify key bacterial indicator taxa across groups. Detrended Correspondence Analysis (DCA) was also employed to explore microbial community structure across samples. Rarefaction method was adopted to extract certain number of sequences from each sample pool to reach the same depth, to predict the number and abundance of ASVs observed by each group at that sequencing depth.

### 2.6 Statistical Analysis

Physicochemical data were analysed using one‐way analysis of variance (ANOVA) to test for differences among treatments. Mean separation was performed using Tukey's Honest Significant Difference (HSD) test at a 95% confidence level (*p* < 0.05). All statistical analyses were conducted using SAS software (version 9.1). The sequence data was analysed using the QIIME 2 software pipeline with default settings. Richness and diversity indexes (Shannon‐Weaver, Evenness index and Chao 1) were performed for each pre‐processed data set using the R version 3.4.1. The Principal Component Analysis (PCA) and Principal Coordinates Analysis (PCOA) were carried out using Canoco version 5.0.

## 3 Results

### 3.1 Soil Physicochemical Properties Under Organic Amendments

The physicochemical properties of soils evaluated under different organic fertilisation treatments, as presented in Table 1, show relative stability in the soil textural fractions with minor variations across the treatments except for silt contents, which are higher in the bulk soil (6.00%). Available phosphorus (P) was significantly enriched under poultry manure treatment (34.17 mg/kg) and cattle dung (27.88 mg/kg) relative to both the control (15.12 mg kg^−1^) and bulk soil (10.20 mg kg^−1^). Sodium (Na) concentrations were markedly higher in the control (44.03 mg/kg; 0.19 cmol/kg) compared with bulk and organic treatments (~25–28 mg kg^−1^; 0.11–0.12 cmol kg^−1^). Potassium (K) was highest in cattle dung treatment (21.00 mg/kg; 0.53 cmol/kg) followed by control (17.98 mg/kg; 0.43 cmol/kg), while lower values were recorded in poultry manure and bulk soils at 13.00 and 12.92 mg kg^−1^, respectively. Similarly, cattle dung improved magnesium (Mg) (23.99 mg/kg; 1.91 cmol/kg), while poultry manure contributed the least (14.02 mg/kg; 1.17 cmol/kg). In addition, poultry manure had the highest calcium (Ca) content, with values of 48.01 mg kg^−1^ (2.40 cmol kg^−1^), followed by cattle dung (46.95 mg/kg; 2.32 cmol/kg), while bulk soil had the lowest concentration at 32.94 mg/kg; 1.61 cmol/kg (Table 1).

**TABLE 1:** The physical and chemical properties of the rhizosphere and bulk soil samples.

Analyte	Cattle dung	Poultry manure	Bulk	Control
Clay (%)	18.11 ± 0.18b	18.05 ± 0.32c	16.17 ± 0.15d	18.16 ± 0.12a
Sand (%)	77.98 ± 0.07a	77.98 ± 0.08a	78.06 ± 0.04a	77.97 ± 0.11a
Silt (%)	4.01 ± 0.09b	4.01 ± 0.03b	6.00 ± 0.10a	3.90 ± 0.12b
P (mg/kg)	27.88 ± 0.28b	34.17 ± 0.31a	10.20 ± 0.23d	15.12 ± 0.32c
Na (mg/kg)	28.11 ± 0.14b	27.98 ± 0.07b	25.13 ± 0.12c	44.03 ± 0.31a
K (mg/kg)	21.00 ± 0.11a	13.00 ± 0.11c	12.92 ± 0.16d	17.98 ± 1.03b
Ca (mg/kg)	46.95 ± 0.20b	48.01 ± 0.04a	32.94 ± 0.06d	41.94 ± 0.14c
Mg (mg/kg)	23.99 ± 0.06a	14.01 ± 0.08d	16.64 ± 0.40c	22.62 ± 0.42b
pH	7.29 ± 0.02a	7.25 ± 0.24b	7.27 ± 0.32b	7.79 ± 0.04a
Na (cmol/kg)	0.12 ± 0.00b	0.12 ± 0.00b	0.11 ± 0.00c	0.19 ± 0.00a
K (cmol/kg)	0.53 ± 0.00d	0.34 ± 0.00b	0.32 ± 0.00a	0.43 ± 0.00c
Ca (cmol/kg)	2.32 ± 0.00b	2.40 ± 0.01a	1.61 ± 0.01d	2.04 ± 0.00c
Mg (cmol/kg)	1.91 ± 0.08a	1.17 ± 0.02c	1.82 ± 0.05b	1.82 ± 0.00b
EC (mS/m)	48.41 ± 0.02d	48.98 ± 0.10c	58.95 ± 0.10a	49.01 ± 0.19b
NH4 + N (mS/m)	8.98 ± 0.19a	6.09 ± 0.20b	6.72 ± 0.04c	5.18 ± 0.14d
NO3‐N (mg/kg)	11.08 ± 0.29c	13.25 ± 0.05a	5.84 ± 0.18d	12.09 0.11b

*Note:* Means with different letters across the row are significantly (*p* < 0.05) different from one another.

### 3.2 Microbial Community Composition

**FIGURE 2:**
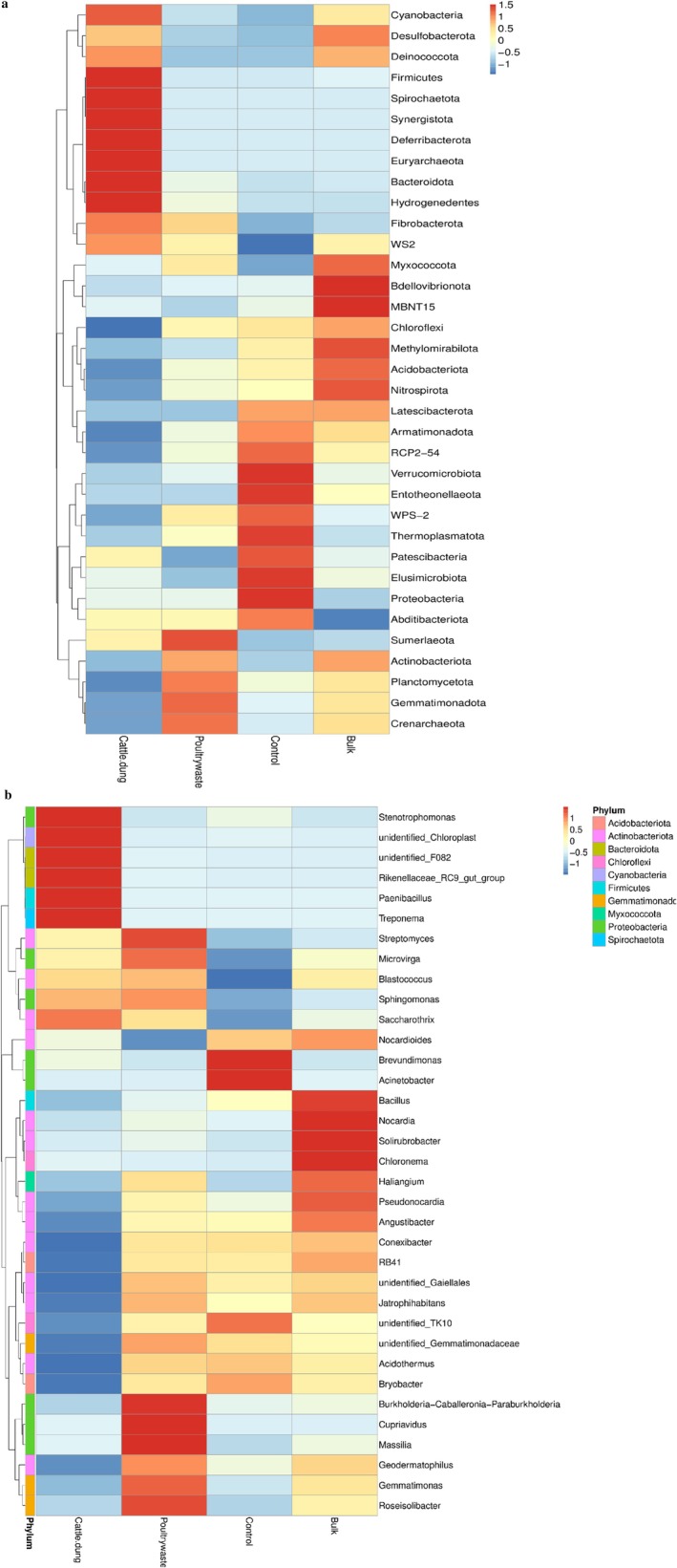
The heatmap representation of the relative abundances of the bacteria at (a) phylum and (b) genus levels, across the treatments.

The heatmap at the phylum level in Figure 2 highlights variation in microbial communities across fertilisation treatment. Cattle manure amendment showed a higher abundance of Cyanobacteria, Firmicutes, Spirochaeota, Synergistota, Euryarchaeota, Bacteroidota and Hydrogenedentes. In poultry manure treatment, the most abundant bacterial phyla include Sumeriaota, Gemmatimonadota, Crenarchaeota, Planctomycota and Actinobacteriota. The untreated control was characterised by Verrucomicrobiota, Entotheonellaeota, Thermoplasmatota, Elusimicrobiota and Proteobacteria; also in the bulk soil, Bdellovibrionota, MBNT15, Methylomirabilota, Nitrospirota (Figure 2a).

At genus level, the most abundant bacterial communities across treatments include Cattle dung; Stenotrophomonas, Unidentified chloroplast, Unidentified F082, Rikenellaceae, Paenibacillus and Treponema, Poultry manure; Burkholderia (Paraburkholderia), Cupriavidus, Massilia, Roseisolibacter, Gemmatimonas and Geodermatophilus, while the untreated control included Brevundimonas and Acinetobacter and bulk soil showed higher abundance of Bacillus, Nocardia, Solirubrobacter, Chloronema and Pseudonocardia (Figure 2b).

The scale bar represents a colour saturation gradient based on the relative abundances of the genera that have been *z*‐score converted.

**FIGURE 3:**
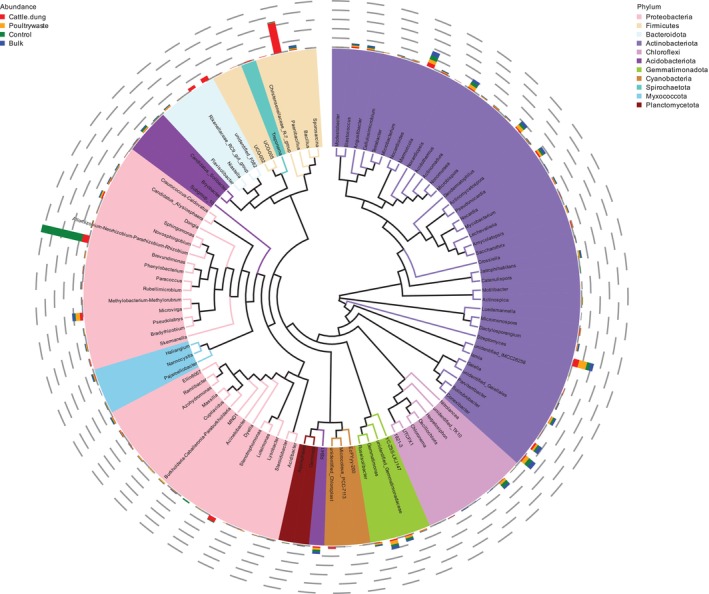
Phylogenetic representation of bacterial genera in the soybean rhizosphere under different organic fertilisation treatments.

Figure 3 shows LEfSe analyses with significant taxa from each treatment. Among these taxa were those in the phylum Proteobacteria, such as Pseudomonas, Acinetobacter and Sphingomonas and Firmicutes, such as Paenibacillus and Clostridium, clearly associated with the organically amended soils. Treatments with poultry manure had higher numbers of members of Actinobacteria, such as Streptomyces and Micromonospora, while cattle dung had supporting taxa such as Rubellimicrobium and Phenylobacterium. The bulk soil had diverse Acidobacteria and Chloroflexi groups. Meanwhile, control soils had members from core taxa such as Gammaproteobacteria, among others.

**FIGURE 4:**
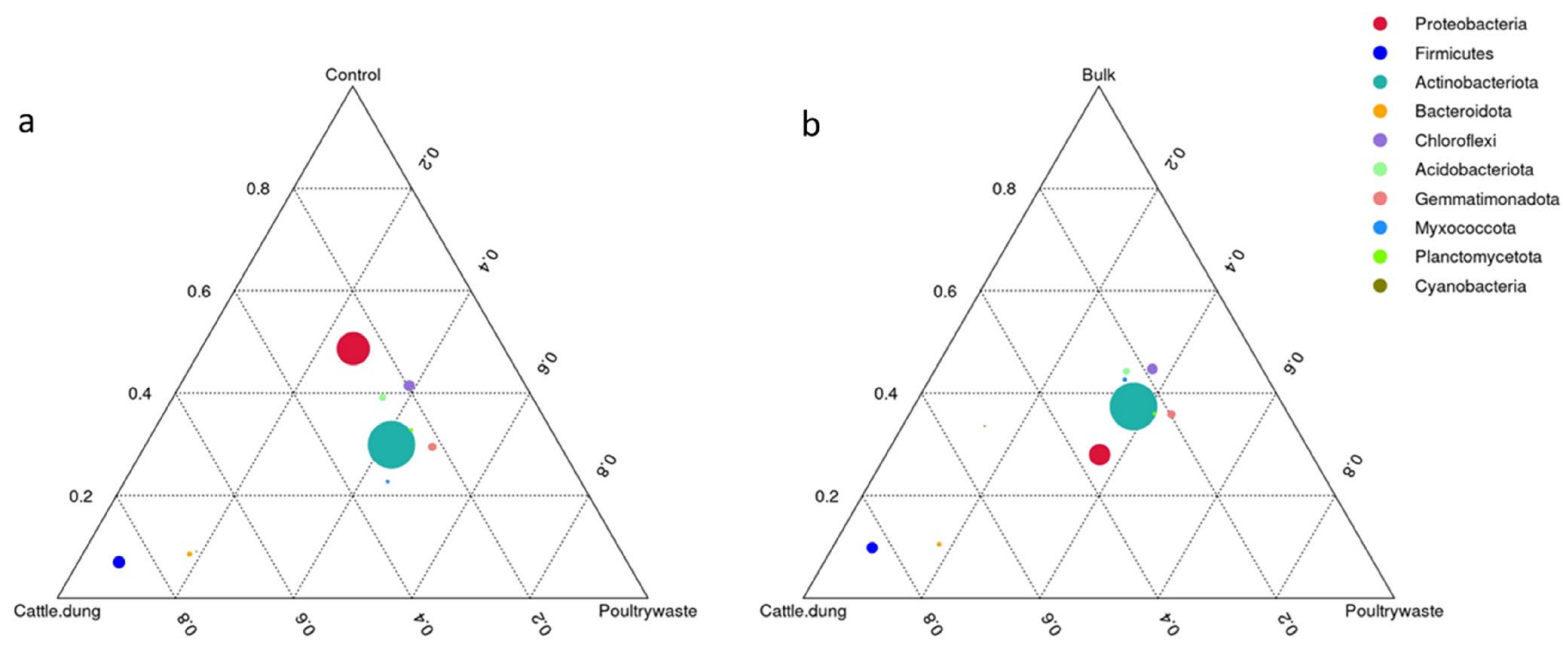
Ternary distribution of dominant bacterial phyla across soil treatments (a) cattle dung, poultry manure and control, (b) cattle dung, poultry manure and bulk soil.

The ternary plots uncovered specific distinctions within treatment combinations regarding the distribution of dominant bacterial phyla (Figure 4) in the comparison of cattle dung, poultry manure and control (untreated soils) (Figure 4a). Proteobacteria and Planctomycetota were more evenly distributed but showed stronger associations with control and poultry manure treatments. Firmicutes cluster toward cattle dung, while Acidobacteriota and Chloroflexi are closer to the control, implying their predominance in unamended soils. When cattle dung, poultry manure and bulk soils are compared (Figure 4b), Planctomycetota were found to be strongly enriched under poultry manure treatment, with Firmicutes consistently associated with cattle dung. Among these conditions, bulk soils were characterised by the presence of oligotrophic phyla such as Acidobacteriota and Chloroflexi. Proteobacteria appeared broadly distributed across all treatments.

### 3.3 Diversity Metrics

#### 3.3.1 Alpha Diversity of Bacterial Communities

**FIGURE 5:**
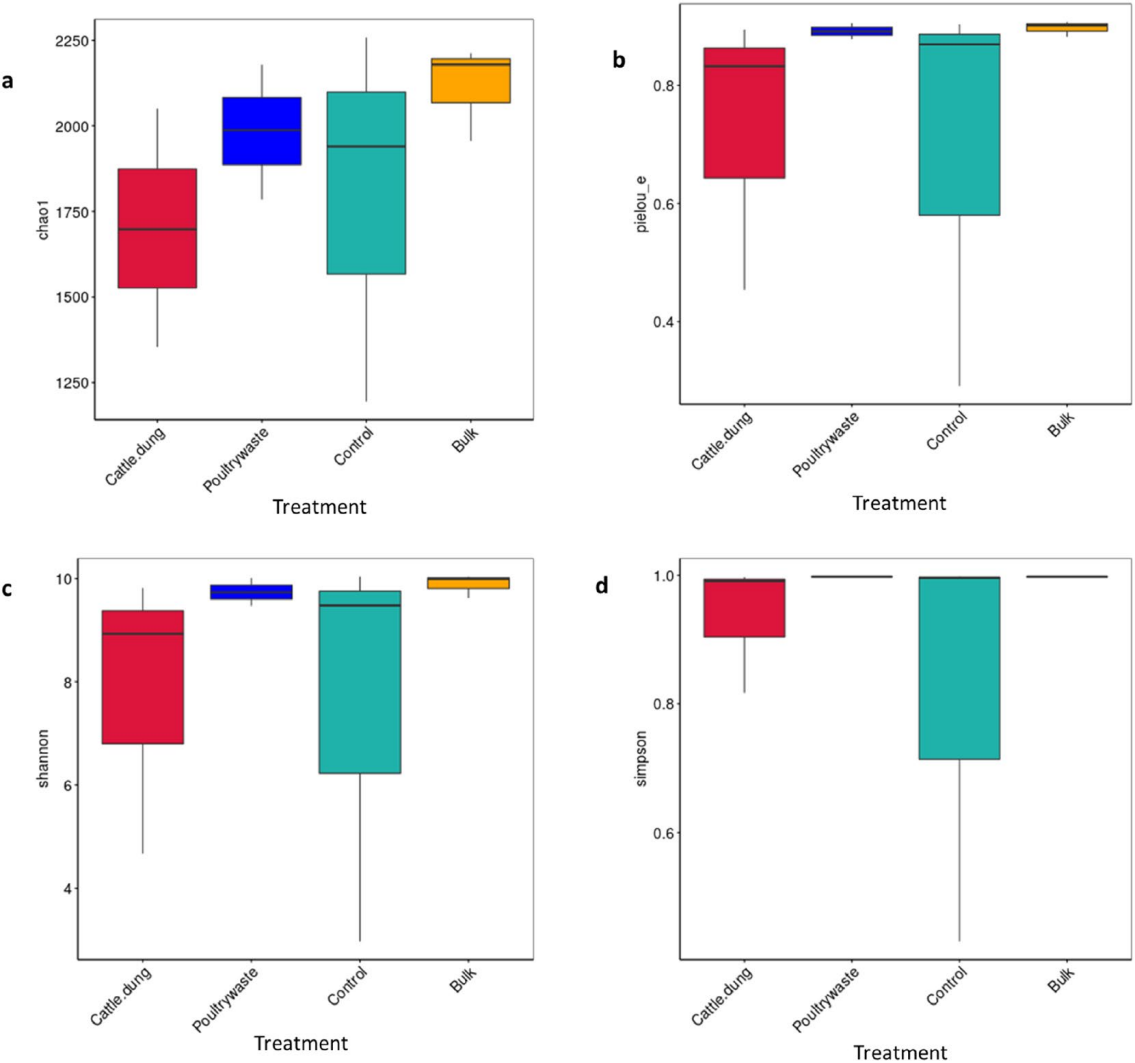
The boxplot of the bacteria community indices of soybean rhizosphere showing the (a) Chao1 (b) Pielou_e (c) Shannon and (d) Simpson.

Alpha diversity analysis revealed significant differences in bacterial richness and evenness among treatments (Table 2, Figure 5). Bulk soil exhibited the highest richness, with Chao1 richness (5034.16) and observed features (4943), which was higher than that of other treatments. This was closely followed by poultry manure (Chao1: 4615.98; observed: 4529). Cattle dung recorded the lowest richness (Chao1: 3936.10; observed: 3867). This trend was also observed in the boxplots, where the Chao1 estimates indicated the bulk and control soils as richer than poultry manure and cattle dung (Figure 5a). Diversity indices followed a similar pattern; this further revealed that poultry manure (Shannon: 10.465; Simpson: 0.998) and bulk soil (Shannon: 10.692; Simpson: 0.998) showed the most diverse and evenly distributed bacterial communities (Figure 5c,d), as also reflected by higher Pielou's evenness (0.862 and 0.871 respectively) (Figure 5b). On the other hand, control plots exhibited lower diversity (Shannon: 8.671; Simpson: 0.936) and evenness (0.716), with a higher dominance score (0.064).

**TABLE 2:** Alpha diversity indices of soybean rhizosphere treatment.

Treatments	Chao1	Dominance	Goods coverage	Observed features	Pielou_e	Shannon	Simpson
Cattle dung	3936.102	0.022	0.998	3867	0.743	8.853	0.978
Poultry manure	4615.979	0.002	0.998	4529	0.862	10.465	0.998
Control	4475.974	0.064	0.998	4408	0.716	8.671	0.936
Bulk	5034.159	0.002	0.998	4943	0.871	10.692	0.998

#### 3.3.2 Beta Diversity and Community Dissimilarity

**FIGURE 6:**
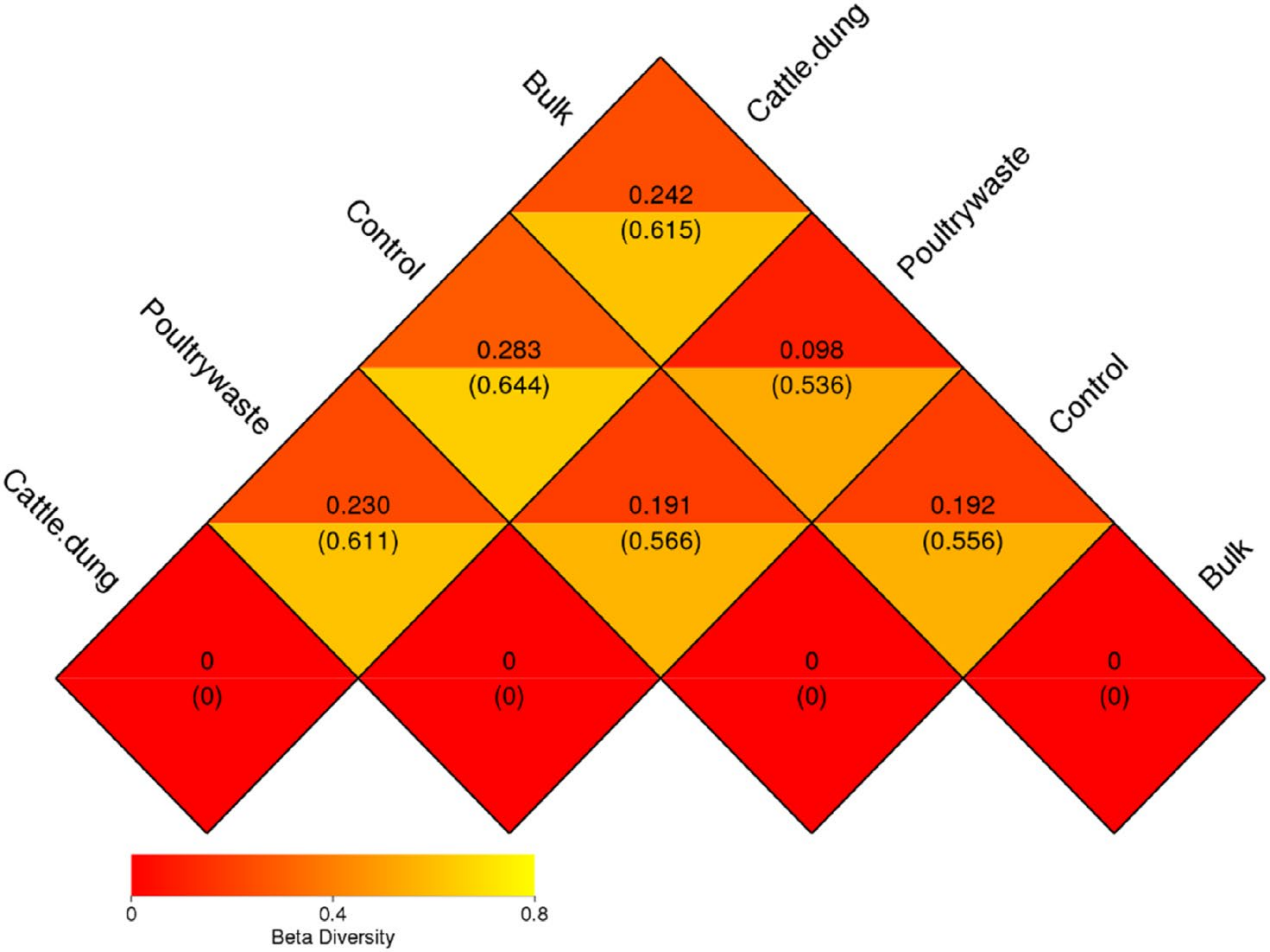
Beta diversity of the bacterial community of soybean rhizosphere.

Pairwise beta diversity comparisons revealed variation in the composition of the bacterial community among the different treatments (Figure 6). Beta diversity values represent the degree of dissimilarity among various treatments, with corresponding statistical values that indicate the strength of these differences. The lowest dissimilarity was recorded between poultry manure and control treatments (*β* = 0.098, stat = 0.536), indicating high similarity between the bacterial communities in these two groups. Cattle dung treatments and poultry manure treatments (*β* = 0.283, stat = 0.644) were the most divergent from each other with respect to microbial composition. Moderate dissimilarities were recorded between bulk and control (*β* = 0.192, stat = 0.556) and between cattle dung and control (*β* = 0.191, stat = 0.566), as well as bulk vs. cattle dung comparison (*β* = 0.242, stat = 0.615).

#### 3.3.3 ASV Overlap and Core Microbiome

**FIGURE 7:**
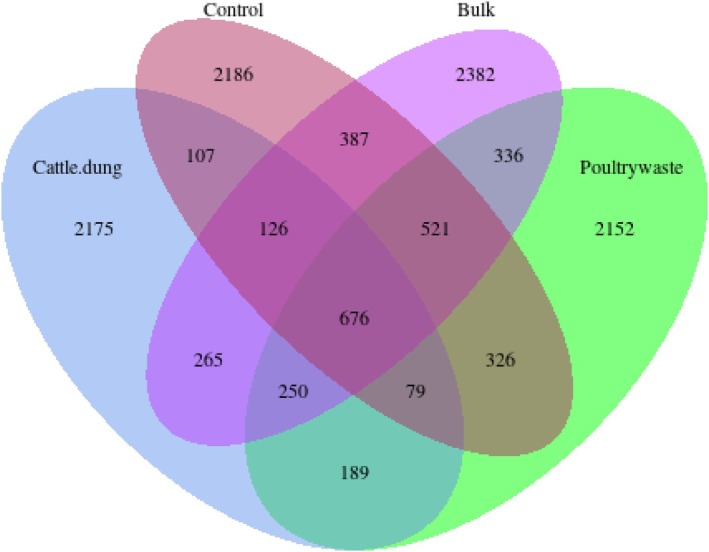
Venn diagram showing shared and unique amplicon sequence variants (ASVs) across cattle dung, poultry manure, control and bulk soil treatments, highlighting both treatment‐specific microbial assemblages and a conserved core microbiome.

According to the Venn diagram, the amplicon sequence variants (ASVs) specific to each treatment, namely cattle dung, poultry manure, bulk soil and control, were identified as both unique and overlapping. More specifically, bulk soil (2382 ASVs), control (2186 ASVs), cattle dung (2175 ASVs) and poultry manure (2152 ASVs) showed high numbers of unique ASVs, indicating the specific microbial assemblages for each treatment. The presence of 676 shared ASVs across all treatments indicates a stable core microbiome, while the high number of unique ASVs in each treatment reflects strong selective effects of organic amendments on rhizosphere microbial assembly. Pairwise overlap differs, with 326 ASVs being common between poultry manure and bulk soil; 265 between cattle dung and bulk soil; and 250 between cattle dung and poultry manure. Comparatively fewer ASVs (107–387) were common to the control and amended plots (Figure 7).

### 3.4 Multivariate Analysis of Microbial Community Structure

**FIGURE 8:**
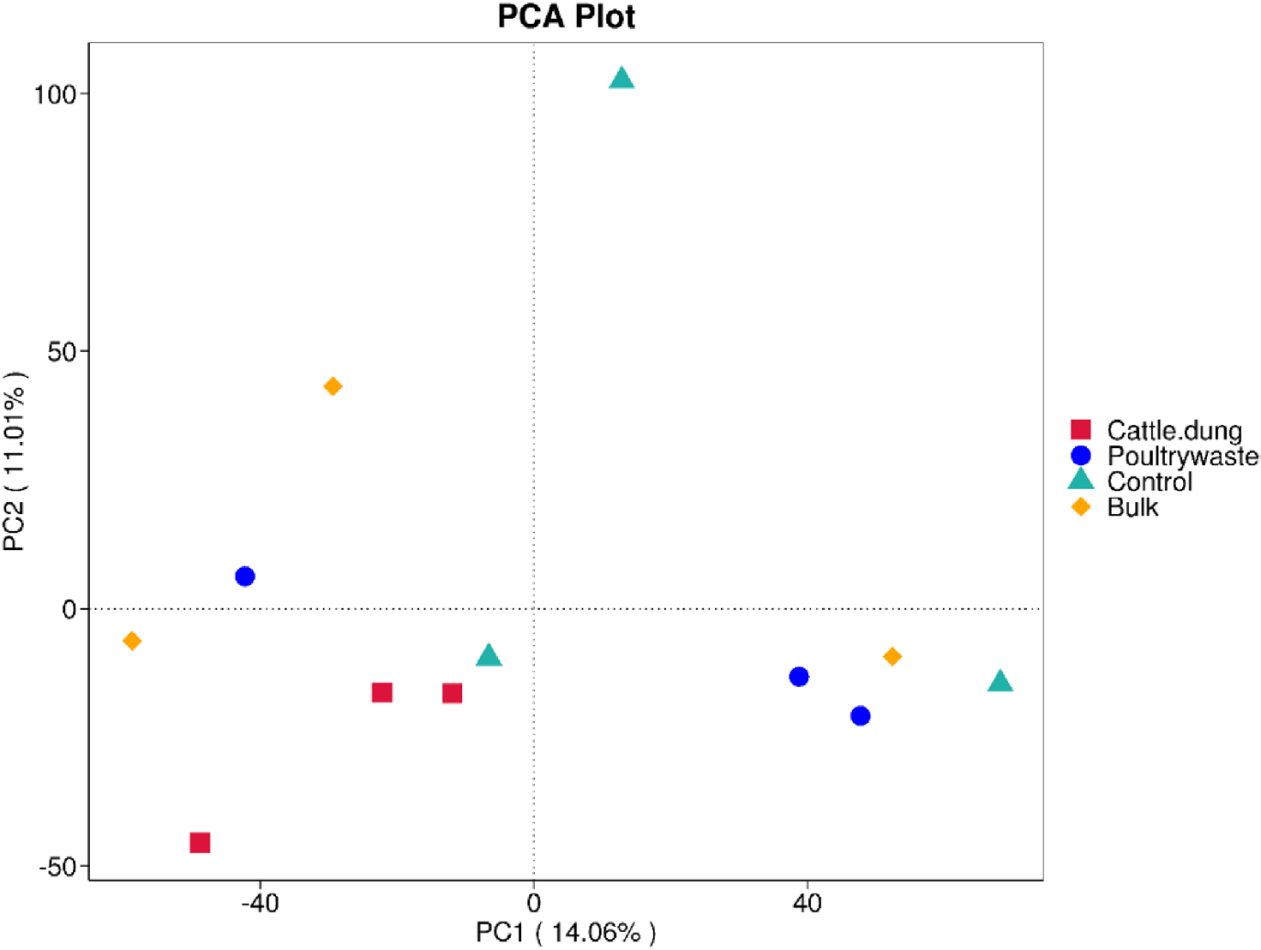
Principal component analysis (PCA) plot of bacterial community distribution across the treatments.

Principal component analysis (PCA) visualised the differences in the bacteria community structures across the treatments. The first two components, PC1 (14.06%) and PC2 (11.01%) explained 25% of the total variance in community composition. Clear clustering patterns were observed between treatments. On the negative axis of PC1, cattle dung samples clustered distinctly, indicating the uniqueness of these microbial assemblages compared with other treatments. Furthermore, poultry manure samples were separated toward the positive side of PC1, indicating that poultry manure supported a divergent community structure. Samples of bulk soil, on the other hand, were more dispersed along both axes, thereby revealing the heterogeneous community composition. However, the control samples were in intermediate positions but exhibited some degree of overlap with poultry manure along PC1, indicating partial similarity between untreated and poultry‐amended soils regarding microbial structural condition (Figure 8).

**FIGURE 9:**
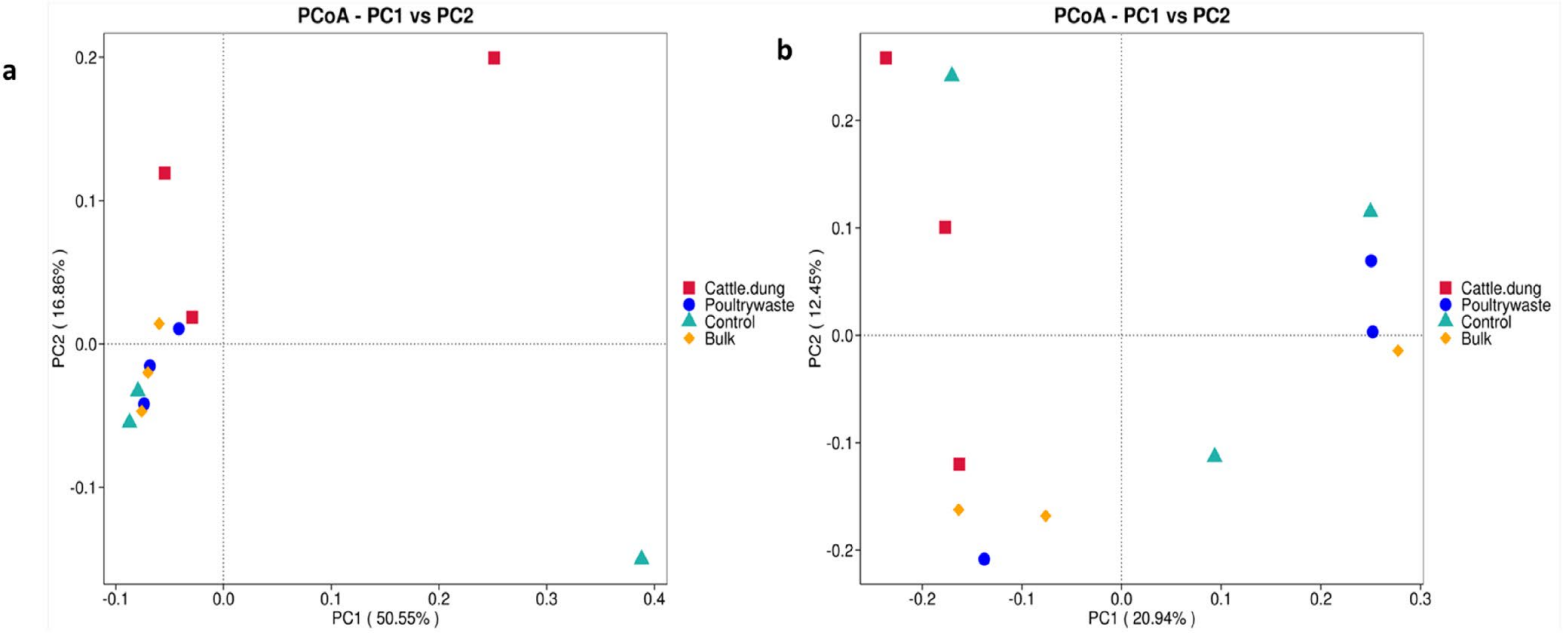
PCoA based on (a) weighted unifrac distance and (b) unweighted unifrac distance.

Principal coordinate analysis (PCoA) based on Weighted UniFrac and Unweighted UniFrac distances was performed to assess treatment‐driven differences in community structure (Figure 9). The Weighted UniFrac plot (Figure 9a) accounted for both the presence and relative abundance of taxa, with both PC1 (50.55%) and PC2 (16.86%) accounting for significant variation in the bacterial communities. The cattle dung samples clustered distinctly along the positive side of PC2, while bulk soil, control and poultry manure samples were more clustered toward the lower side of PC2, indicating differences in dominant taxa influenced by organic inputs. In contrast, the Unweighted UniFrac plot in Figure 9b shows that PC1 showed a low variance contribution (20.94%) and PC2 (12.45%). Cattle dung, poultry manure, bulk and control samples appeared more dispersed, with partial overlaps, but also distinct shifts along both axes. This pattern indicates that while cattle dung strongly influenced abundant taxa (Weighted UniFrac), all treatments altered the presence of rare taxa, leading to broader community‐level differences (Unweighted UniFrac) (Figure 9).

The pairwise beta‐diversity pattern comparisons showing Multi‐Response Permutation Procedure (MRPP) results among the cattle dung, poultry manure, control and bulk soil treatments revealed no statistically significant differences in microbial community structure. Although the *R*‐value between cattle dung and poultry manure was relatively high (0.727), the difference was not statistically significant (*p* = 0.20), indicating that observed variation may be due to random effects. Cattle dung vs. bulk soil (*R* = 0.111, *p* = 0.20) and cattle dung vs. Unamended control (*R* = 0.074, *p* = 0.50) exhibited minimal differentiation from the control (*R* = −0.148, *p* = 0.90) and bulk soil (*R* = 0.037, *p* = 0.30), indicating high compositional similarity (Table 3).

**TABLE 3:** Multi‐response permutation procedure (MRPP) analysis result.

Group	*A*	Observed‐delta	Expected‐delta	*R*‐Value	*p*‐Value
Cattle.dung‐Poultry manure	0.04678	0.69325	0.72727	0.33333	0.2
Cattle.dung‐Control	0.02004	0.81307	0.82969	0.07407	0.5
Cattle.dung‐Bulk	0.01639	0.71935	0.73134	0.11111	0.2
Poultry manure‐Control	−0.0026	0.72639	0.72453	−0.1482	0.9
Poultry manure‐Bulk	−0.0157	0.63267	0.62286	0.03704	0.3
Control‐Bulk	−0.0213	0.75249	0.73682	0.18519	0.9

### 3.5 Functional Prediction (COG and Pathways)

#### 3.5.1 Predicted Functional Profiles of the Rhizosphere Microbiome

**FIGURE 10:**
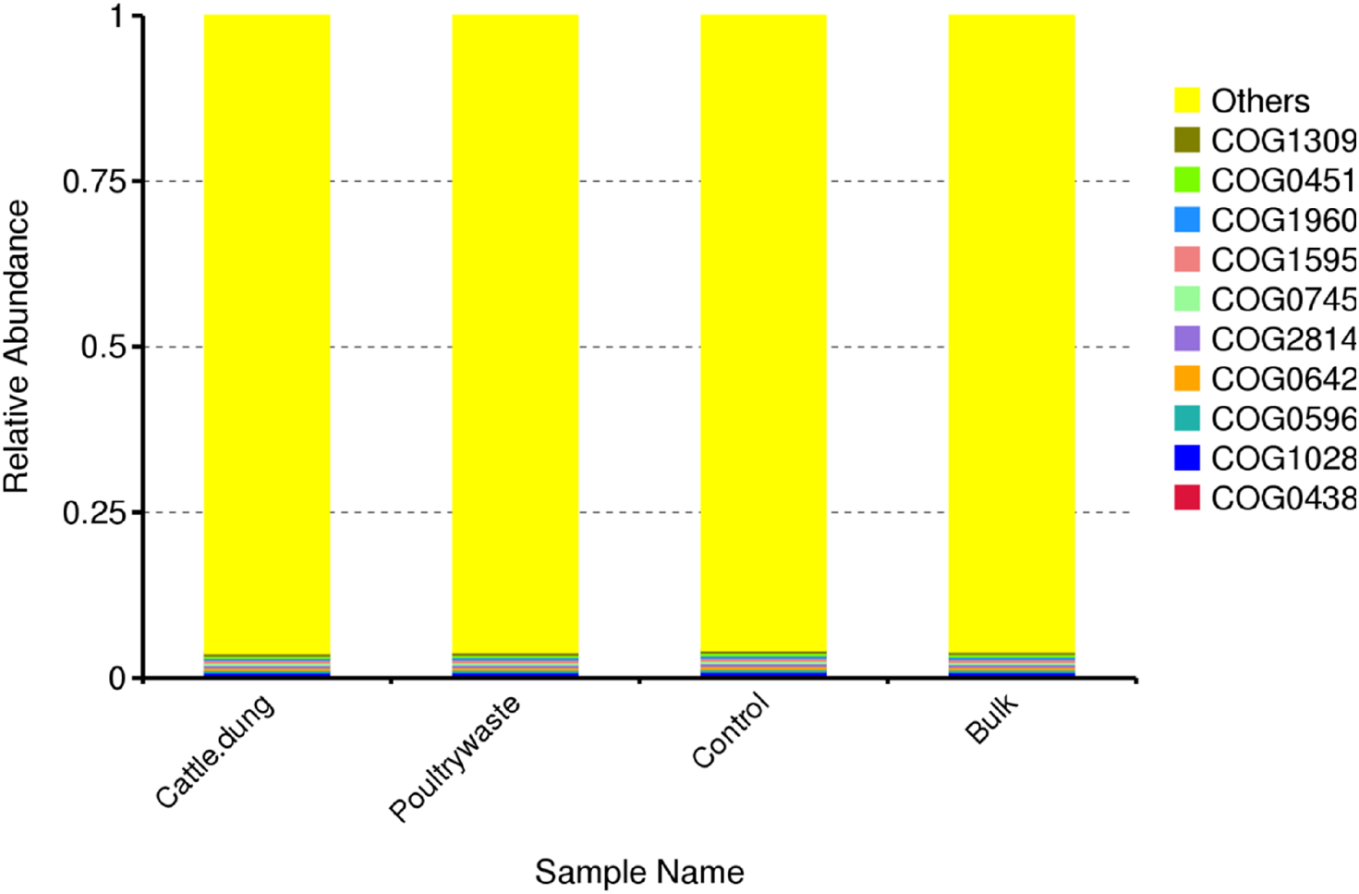
Clusters of Orthologous Groups (COG) of proteins across the organic amendment of soybean rhizosphere and controls.

Similar relative abundance of the functional profiling was recorded across the treatments based on COG categories of the 10 most abundant functions distributed across the major categories. This includes information storage and processing (COG1309, COG0451, COG0438), cellular processes and signalling (COG0745, COG2814, COG0596) and metabolism (COG1960, COG1595, COG0642, COG1028) (Figure 10).

**FIGURE 11:**
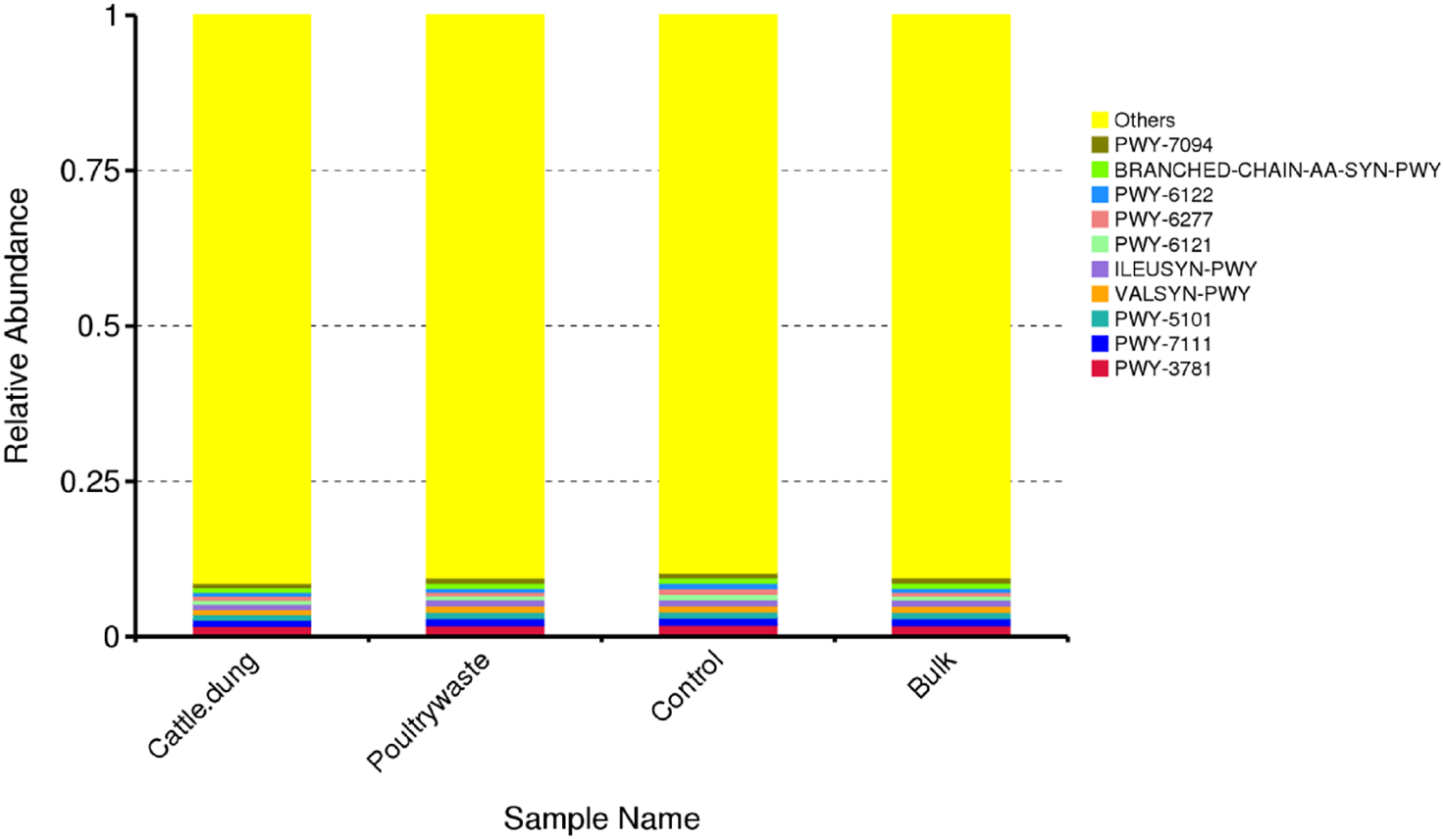
Relative abundance of predicted MetaCyc metabolic pathways across the treatments.

The most abundant functional profile based on the predicted metabolic pathways (MetaCyc identifiers) includes branched‐chain amino acid biosynthesis pathways (e.g., BRANCHED‐CHAIN‐AA‐SYN‐PWY, ILEUSYN‐PWY, VALSYN‐PWY), as well as the amino acid and central metabolic routes (e.g., PWY‐7094, PWY‐6122, PWY‐6277, PWY‐6121, PWY‐5101, PWY‐7111, PWY‐3781). While the microbial communities differ in their taxonomic composition, the core metabolic functions, especially amino acid biosynthesis, remain conserved across various organic amendments and control soils (Figure 11).

#### 3.5.2 Functional Gene Annotation and Abundance

**FIGURE 12:**
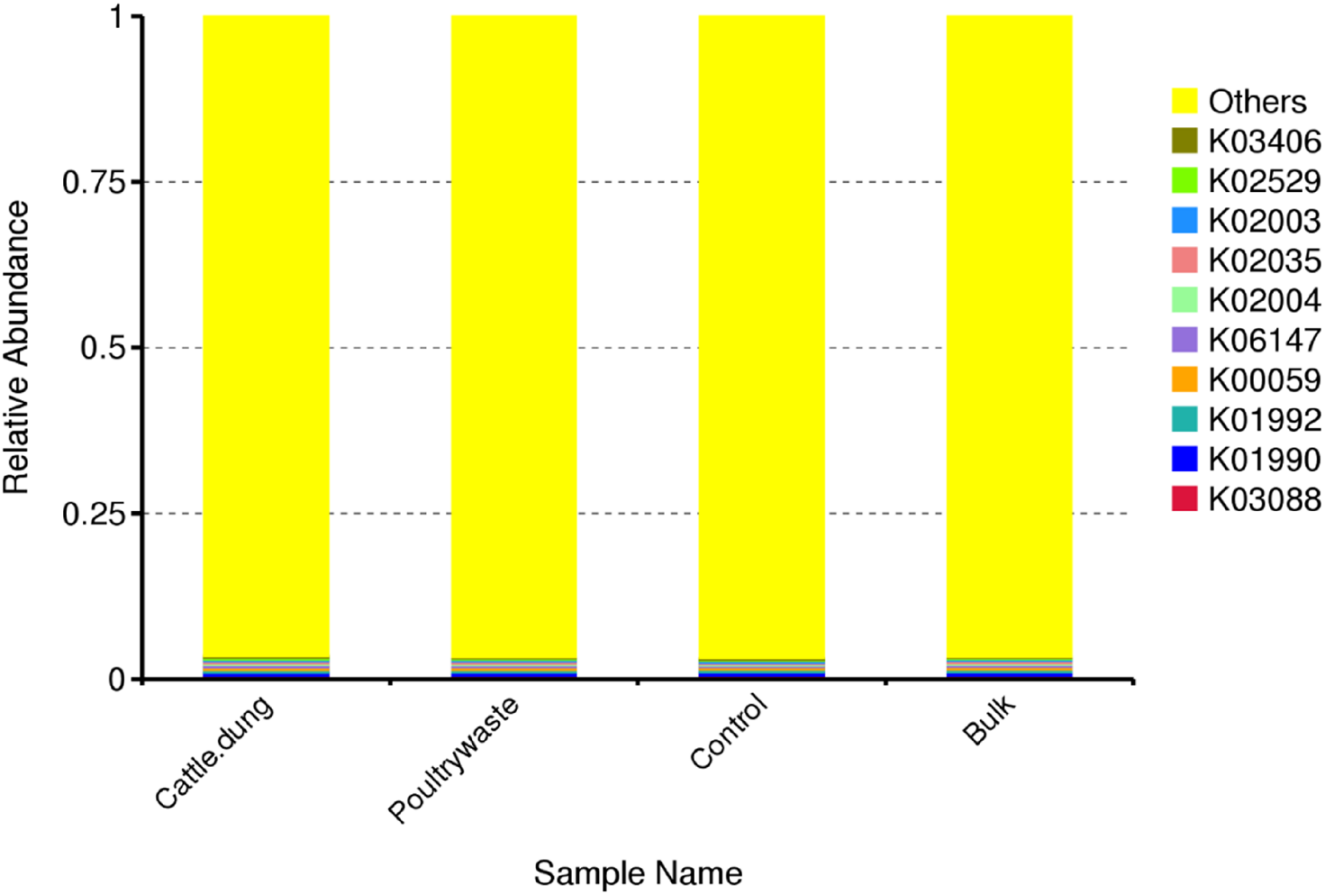
KEGG orthologs of the relative abundance of functional genes across the treatments.

This bar chart showed KEGG orthologs (KOs) predicted over various treatments such as cattle dung, poultry manure, control and bulk soil. Among the specifically annotated KOs, a minor fraction (< 5%) was responsible for the total relative abundance and these include some functions on treatment. Among the individually annotated KOs, a very few of them (< 5%) contributed to the overall relative abundance. These functioned for metabolism and biosynthesis: K00059 (acetyl‐CoA synthetase), K02003/K02004/K02035 (ABC transporters), K02529 (ATP synthase subunits), genetic information processing: K01990, K01992 (ribosomal proteins), K03088 (transcription initiation factor) and cellular processes and signalling: K03406 (two‐component system sensor kinase), K06147 (membrane‐associated proteins) (Figure 12).

The top 10 Pfam families were classified into three functional categories: transport proteins (PF00005, PF02518, PF00534, PF07690, PF13561), signal transduction (PF00072, PF00512, PF04542) and DNA replication/repair (PF00271, PF07992) (Figure S1).

The top 10 functional proteins from The Institute for Genomic Research Protein Families (TIGRFAM) protein families were grouped into three functional categories. Transport‐related proteins included an efflux transporter membrane fusion protein (TIGR01730). Transcriptional regulation was represented by an ECF‐type sigma factor (TIGR02937), while TIGR00231 was associated with GTP‐binding proteins involved in translation (Figure S2).

#### 3.5.3 Shared and Unique Functional Attributes Across Treatments

Based on Weighted UniFrac distances, differences in microbial community composition are driven by the organic amendments. Cattle dung treatment forms a separate cluster, implying a unique microbial assemblage that is likely influenced by the nutrient‐rich and diverse organic inputs in cattle dung manure. The control treatment clusters closely with the poultry manure and bulk soil, reflecting more similar community structures and potentially less microbial enrichment compared to cattle dung amendment.

At the phylum level, Proteobacteria dominated across all treatments, followed by Firmicutes, Actinobacteriota and Bacteroidota, suggesting their key role in soil nutrient cycling (Supplementary Figure 3). Genera such as UTCFX1, Virgibaculum, Turicibacter, Clostridium sensu stricto 1 and unidentified‐Chloroplast exhibited higher relative abundances in the cattle dung treatment, suggesting that cattle dung promotes bacterial groups involved in organic matter degradation, fermentation and nutrient mineralisation.

Conversely, Leifsonia, Dyella, Acidipillium and Ilumatobacter were more enriched in poultry manure treatment, reflecting microbial adaptation to nutrient‐rich, nitrogen‐dense conditions typical of poultry manure (Figure S4a). Overall, bulk soil exhibited a higher abundance and diversity of bacterial genera compared to the control, indicating a richer and more active microbial community. Genera such as Sporosarcina, Herbiconiux, Aeromicrobium, Saccharomonospora, Actinophytocola, Amnibella, Parviterribacter and Saccharothrix were markedly enriched in bulk soils. These bacteria are known for their roles in organic matter decomposition, nutrient cycling and soil structure stabilisation.

Conversely, the control soils displayed lower relative abundances across most genera, reflecting limited nutrient availability and reduced microbial proliferation. The genus Luteolibacter was relatively more represented in control samples, possibly suggesting adaptation to nutrient‐limited or stress‐prone conditions (Figure S4b).

**FIGURE 13:**
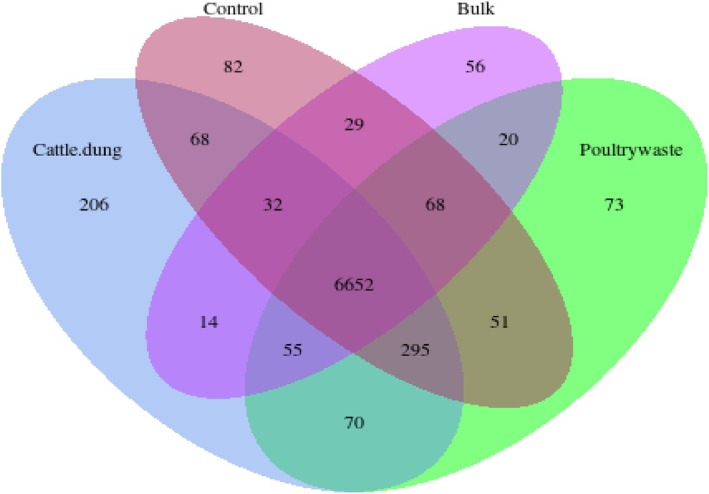
Venn diagram of PICRUSt2 results of microbial ASVs across treatments.

The Venn diagram illustrates the distribution of microbial ASVs across four soil treatments, control, bulk, cattle dung and poultry manure. A total of 6652 ASVs were common to all treatments, indicating a stable core microbiome. Cattle dung showed the highest number of unique ASVs (206). Poultry manure, control and bulk treatments had 73, 82 and 56 unique ASVs, respectively. There were also several shared ASVs among different combinations of treatments, with 295 ASVs shared between cattle dung and poultry manure, highlighting partial overlap in their microbial influence (Figure 13).

## 4 Discussion

This investigation profiled the bacterial diversity and functional dynamics of the soybean ( *Glycine max* L.) rhizosphere under various organic fertilisation, providing insights into how amendments with cattle dung and poultry manure affect soil nutrient status, microbial community composition and functional potential. The results highlighted both taxonomic shifts and functional stability in the rhizosphere microbiome, pointing to the ecological resilience of soil microbial communities under organic management. Plant growth and soil microbial diversity are influenced by soil nutrient availability and structure (Chen et al. 2024; Dlamini et al. 2022). The application of organic amendments markedly enhanced soil nutrient availability, with poultry manure contributing higher levels of available phosphorus and calcium, whereas cattle dung enriched the potassium and magnesium contents. These results are consistent with the reports that poultry manure, due to its rapid decomposition and nutrient solubility, has a high capacity to enrich P and Ca in soil (Chukwuma et al. 2024; Joseph et al. 2025). In contrast, the fibrous nature of the cattle manure allows for gradual release of nutrients, thereby maintaining K and Mg availability over time (Ogumba et al. 2024). Interestingly, sodium concentrations in control soils were higher than in amended plots, implying that organic inputs play a role in moderating sodium accumulation and mitigating the risk of sodicity (Singh et al. 2022). The observed shifts in microbial composition correspond closely to changes in soil physicochemical properties. For instance, increased phosphorus and calcium in poultry manure‐amended soils likely supported the proliferation of copiotrophic taxa such as Burkholderia and Cupriavidus, which are typically associated with nutrient‐rich environments and phosphorus cycling (Ayangbenro et al. 2022; Babalola and Adedayo 2023). Conversely, the enrichment of potassium and magnesium in cattle dung treatments aligns with the dominance of decomposer taxa such as Paenibacillus, reflecting adaptation to more complex organic substrates and involvement in organic matter degradation and nutrient mineralisation (Cao et al. 2024; Rastogi et al. 2023). Functionally, the persistence of amino acid biosynthesis and ABC transporter pathways suggests that nutrient acquisition and microbial growth processes may remain stable despite taxonomic restructuring (Enagbonma et al. 2019; Sauma‐Sánchez et al. 2024). These functions are directly linked to nutrient turnover and rhizosphere competence, indicating potential indirect implications for plant nutrient availability and soil fertility (Dlamini et al. 2022). These observations further emphasize the role of organic amendment in enhancing soil chemical quality, which may sustain legume productivity and nodulation efficiency.

Consistent with these physicochemical changes, the 16S rRNA gene sequencing results revealed that the dynamics of the bacterial community in the rhizosphere were influenced by both the treatments and growth stages. Organic inputs distinctly influenced bacterial community structure, favouring divergent assemblages depending on the type of amendment. The cattle dung supported higher relative abundances of Firmicutes, Bacteroidota and Spirochaetota, while poultry manures enriched Proteobacteria, Actinobacteria and Planctomycota. These differences reflect the biochemical properties of the organic amendments, where fibrous substrates in cattle dung favour taxa capable of polymer degradation, while nutrient‐rich poultry manure selects for copiotrophic bacteria (Babalola et al. 2025).

At the genus level, the bacteria *Paenibacillus* and *Treponema*, promoted by cattle dung, were reported to be involved in organic matter decomposition and nitrogen fixation (Cao et al. 2024). Poultry manure enriched beneficial taxa such as *Burkholderia* (*Paraburkholderia*) and *Cupriavidus*, well documented for their plant growth–promoting traits and phosphorus solubilisation (Babalola and Adedayo 2023). Contrarily, the untreated control favoured opportunistic genera such as Acinetobacter and Brevundimonas. These results suggest potential benefits to plant growth and soil health, which require validation through plant performance studies. However, a proportion of ASVs remained unclassified at lower taxonomic levels, which is a common limitation of 16S rRNA‐based profiling due to database constraints and the presence of novel or poorly characterised soil taxa. The interpretation was therefore focused primarily on well‐resolved taxa with known ecological functions (Douglas et al. 2020; Matchado et al. 2024). Although plant growth parameters were not measured, the enrichment of plant growth–promoting taxa (e.g., Burkholderia, Paenibacillus) and functional pathways related to nutrient metabolism suggests potential agronomic benefits; however, these require validation in future studies integrating plant performance metrics.

Alpha diversity indices revealed greater richness and evenness in bulk soils and poultry manure treatments compared with cattle dung, which supported comparatively less diverse communities. This agrees with observations that poultry manure creates nutrient‐rich environments conducive to diverse microbial proliferation (Ayangbenro et al. 2022). Cattle dung, on the other hand, appears to favour a more specialised microbial consortium that is more specialised in degrading recalcitrant organic matter. Bulk soils supported the highest richness, likely due to the presence of oligotrophic phyla such as Acidobacteria and Chloroflexi, which thrive under resource‐limited conditions (Jia et al. 2024). Beta diversity and ordination analyses indicate strong community divergence between poultry manure and cattle dung treatments, while poultry manure communities overlapped partially with the control. This overlap suggests that poultry manure amendments may enhance microbial diversity without substantially altering baseline soil microbial networks. In contrast, cattle dung appears to promote distinct microbial clustering patterns, reflecting a stronger selective influence on community composition (Liu et al. 2024).

Despite substantial taxonomic variation across treatments, functional profiling indicated conservation of core metabolic processes, particularly amino acid biosynthesis, ABC transporter activity and energy metabolism. This observation is consistent with the principle of functional redundancy, where different microbial taxa perform overlapping ecological functions, thereby contributing to ecosystem stability (Enagbonma et al. 2019). The universal enrichment of branched‐chain amino acid biosynthesis pathways suggests their important role in microbial growth and adaptation, irrespective of community composition. Functional mechanisms of this nature help ensure that soil‐related processes that are essential for nutrient cycling and stress resilience are preserved even as the composition of microbial assemblages changes (Sauma‐Sánchez et al. 2024). Organic amendments shape both soil physicochemical properties and microbial assemblages in the soybean rhizosphere, with implications for soil health and crop productivity. Poultry manure enhances microbial diversity and nutrient enrichment, with the potential to support plant–microbe interactions and improved plant growth. Cattle dung has been shown to sustain less diverse yet distinctive communities, which may contribute to long‐term soil fertility and organic matter turnover. Importantly, core metabolic functions were conserved across treatments, suggesting potential functional redundancy (Adebayo et al. 2025).

In conclusion, this study demonstrates that organic fertilisation significantly alters the taxonomic composition of the soybean rhizosphere microbiome, with poultry manure enhancing microbial diversity and cattle manure promoting specialised decomposer communities. Despite these compositional shifts, core metabolic functions remain relatively stable, suggesting functional resilience within the soil microbiome. However, the study is limited by its reliance on predictive functional profiling and the absence of direct plant performance measurements. Future research should integrate metagenomic approaches and plant growth assessments to validate the functional implications of these microbial shifts. Taken together, these findings highlight the potential of targeted organic fertilisation strategies to modulate rhizosphere microbial communities and improve soil health within sustainable agricultural systems.

## Author Contributions

**Akinlolu Olalekan Akanmu:** software, writing – review and editing, visualization, co‐supervision. **Olubukola Oluranti Babalola:** conceptualization, supervision, funding acquisition, project administration, resources, software, writing – review and editing, formal analysis, visualization. **Ijeoma Emelda Osuji:** investigation, methodology, writing – original draft, writing – review and editing, software, data curation, formal analysis.

## Funding

This research was funded by the International Centre for Genetic Engineering and Biotechnology (ICGEB) (Grant number: CRP/ZAF22‐93) awarded to OOB.

## Conflicts of Interest

The authors declare no conflicts of interest.

## Supporting information


**Figure S1:** Relative abundance of top Pfam families across the treatments.
**Figure S2:** Relative abundance of top TIGRFAM families across the treatments.
**Figure S3:** Unweighted pair‐group method with arithmetic mean (UPGMA).
**Figure S4a:** MetagenomeSeq analysis between groups (Cattle dung‐Poultry manure).
**Figure S4b:** Metagenome Seq analysis between groups (Control‐Bulk).

## Data Availability

The raw sequences have been deposited with NCBI under the BioProject accession number PRJNA1177761.

## References

[emi470370-bib-0001] Adebayo, A. A. , B. J. Enagbonma , and O. O. Babalola . 2025. “Comparative Metagenomics on Community Structure and Diversity of Rhizomicrobiome Associated With Monoculture and Soybean Precedent Carrot.” Scientific Reports 15, no. 1: 28161. 40750823 10.1038/s41598-025-13605-z PMC12317137

[emi470370-bib-0002] Agarwal, P. , R. Vibhandik , R. Agrahari , A. Daverey , and R. Rani . 2024. “Role of Root Exudates on the Soil Microbial Diversity and Biogeochemistry of Heavy Metals.” Applied Biochemistry and Biotechnology 196, no. 5: 2673–2693. 37191824 10.1007/s12010-023-04465-2

[emi470370-bib-0003] Ajiboye, T. T. , A. S. Ayangbenro , and O. O. Babalola . 2022. “Functional Diversity of Microbial Communities in the Soybean ( *Glycine max* L.) Rhizosphere From the Free State, South Africa.” International Journal of Molecular Sciences 23, no. 16: 9422. 36012686 10.3390/ijms23169422 PMC9409019

[emi470370-bib-0004] Akanmu, A. O. , O. M. Olowe , A. T. Phiri , et al. 2023. “Bioresources in Organic Farming: Implications for Sustainable Agricultural Systems.” Horticulturae 9, no. 6: 659.

[emi470370-bib-0005] Alori, E. T. , O. O. Osemwegie , A. L. Ibaba , et al. 2024. “The Importance of Soil Microorganisms in Regulating Soil Health.” Communications in Soil Science and Plant Analysis: 55(17), 2636–2650.

[emi470370-bib-0006] Al‐Salameen, M. A. , M. T. Abdullah , A. M. Morsy , F. A. Aryan , and M. A. Almousa . 2023. “A Preliminary Investigation to Explore the Diversity of Recent Benthic Foraminifera Using Taxonomical and Molecular Approach: A Case Study From Northwestern Part of the Arabian Gulf, Kuwait.” Life Science Journal 20, no. 8: 1–11.

[emi470370-bib-0007] Antonangelo, J. A. , S. Culman , and H. Zhang . 2024. “Comparative Analysis and Prediction of Cation Exchange Capacity via Summation: Influence of Biochar Type and Nutrient Ratios.” Frontiers in Soil Science 4: 1371777.

[emi470370-bib-0008] Ayangbenro, A. S. , C. F. Chukwuneme , M. S. Ayilara , et al. 2022. “Harnessing the Rhizosphere Soil Microbiome of Organically Amended Soil for Plant Productivity.” Agronomy 12, no. 12: 3179.

[emi470370-bib-0009] Ayilara, M. S. , B. S. Adeleke , and O. O. Babalola . 2023. “Bioprospecting and Challenges of Plant Microbiome Research for Sustainable Agriculture, a Review on Soybean Endophytic Bacteria.” Microbial Ecology 85, no. 3: 1113–1135. 36319743 10.1007/s00248-022-02136-z PMC10156819

[emi470370-bib-0010] Babalola, O. O. , and A. A. Adedayo . 2023. “Endosphere Microbial Communities and Plant Nutrient Acquisition Toward Sustainable Agriculture.” Emerging Topics in Life Sciences 7, no. 2: 207–217. 37975608 10.1042/ETLS20230069 PMC10754323

[emi470370-bib-0011] Babalola, O. O. , F. O. Ogundeji , and A. O. Akanmu . 2025. “Dataset of 16S rRNA and ITS Gene Amplicon Sequencing of Celery and Parsley Rhizosphere Soils.” BMC Genomic Data 26, no. 1: 60. 40855532 10.1186/s12863-025-01351-0 PMC12376418

[emi470370-bib-0012] Bokulich, N. A. , and D. A. Mills . 2013. “Improved Selection of Internal Transcribed Spacer‐Specific Primers Enables Quantitative, Ultra‐High‐Throughput Profiling of Fungal Communities.” Applied and Environmental Microbiology 79, no. 8: 2519–2526. 23377949 10.1128/AEM.03870-12 PMC3623200

[emi470370-bib-8002] Bokulich, N. A. , B. D. Kaehler , J. R. Rideout , et al. 2018. “Optimizing taxonomic classification of marker‐gene amplicon sequences with QIIME 2′s q2‐feature‐classifier plugin..” Microbiome 6, no. 1: 90. 29773078 10.1186/s40168-018-0470-z PMC5956843

[emi470370-bib-8003] Bolyen, E. , J. R. Rideout , M. R. Dillon , et al. 2019. “Reproducible, Interactive, Scalable and Extensible Microbiome Data Science using QIIME 2.” Nature Biotechnology 37, no. 8: 852–857. 10.1038/s41587-019-0209-9 PMC7015180 31341288

[emi470370-bib-0013] Bray, R. H. , and L. T. Kurtz . 1945. “Determination of Total, Organic, and Available Forms of Phosphorus in Soils.” Soil Science 59, no. 1: 39–46.

[emi470370-bib-8004] Callahan, B. J. , D. Grinevich , S. Thakur , et al. 2021. “Ultra‐accurate Microbial Amplicon Sequencing with Synthetic Long Reads.” Microbiome 9, no. 1: 130. 34090540 10.1186/s40168-021-01072-3 PMC8179091

[emi470370-bib-0014] Cao, R. , Y. Huang , R. Li , K. Li , Z. Ren , and J. Wu . 2024. “Regulation of Nitrogen Transformation and Microbial Community by Inoculation During Livestock Manure Composting.” Environmental Microbiology Reports 16, no. 2: e13256. 38575150 10.1111/1758-2229.13256 PMC10994714

[emi470370-bib-0015] Caporaso, J. G. , C. L. Lauber , W. A. Walters , et al. 2012. “Ultra‐High‐Throughput Microbial Community Analysis on the Illumina HiSeq and MiSeq Platforms.” ISME Journal 6, no. 8: 1621–1624. 22402401 10.1038/ismej.2012.8 PMC3400413

[emi470370-bib-0016] Chen, Q. , Y. Song , Y. An , Y. Lu , and G. Zhong . 2024. “Soil Microorganisms: Their Role in Enhancing Crop Nutrition and Health.” Diversity 16, no. 12: 734.

[emi470370-bib-0017] Chukwuma, C. , C. Oraegbunam , S. Ndzeshala , et al. 2024. “Phosphorus Mineralization in Two Lithologically Dissimilar Tropical Soils as Influenced by Animal Manure Type and Amendment‐To‐Sampling Time Interval.” Communications in Soil Science and Plant Analysis 55, no. 5: 707–722.

[emi470370-bib-0018] Dlamini, S. P. , A. O. Akanmu , and O. O. Babalola . 2022. “Rhizospheric Microorganisms: The Gateway to a Sustainable Plant Health.” Frontiers in Sustainable Food Systems 6: 925802.

[emi470370-bib-0019] Douglas, G. M. , V. J. Maffei , J. R. Zaneveld , et al. 2020. “PICRUSt2 for Prediction of Metagenome Functions.” Nature Biotechnology 38, no. 6: 685–688. 10.1038/s41587-020-0548-6 PMC7365738 32483366

[emi470370-bib-8001] Dubey, S. , K. Gupta , J. Akhtar , et al. 2021. “Plant quarantine for biosecurity during transboundary movement of plant genetic resources.” Indian Phytopathology 74, no. 2: 495–508.

[emi470370-bib-0020] Enagbonma, B. J. , B. R. Aremu , and O. O. Babalola . 2019. “Profiling the Functional Diversity of Termite Mound Soil Bacteria as Revealed by Shotgun Sequencing.” Genes 10, no. 9: 637. 31450818 10.3390/genes10090637 PMC6770954

[emi470370-bib-0021] Fadiji, A. E. , J. O. Kanu , and O. O. Babalola . 2021. “Impact of Cropping Systems on the Functional Diversity of Rhizosphere Microbial Communities Associated With Maize Plant: A Shotgun Approach.” Archives of Microbiology 203, no. 6: 3605–3613. 33973044 10.1007/s00203-021-02354-y

[emi470370-bib-0022] Gamage, A. , R. Gangahagedara , J. Gamage , et al. 2023. “Role of Organic Farming for Achieving Sustainability in Agriculture.” Farming System 1, no. 1: 100005.

[emi470370-bib-0023] Giagnoni, L. , M. Arenella , E. Galardi , P. Nannipieri , and G. Renella . 2018. “Bacterial Culturability and the Viable but Non‐Culturable (VBNC) State Studied by a Proteomic Approach Using an Artificial Soil.” Soil Biology and Biochemistry 118: 51–58.

[emi470370-bib-0024] Gu, Y. , Y. Liu , J. Li , et al. 2022. “Mechanism of Intermittent Deep Tillage and Different Depths Improving Crop Growth From the Perspective of Rhizosphere Soil Nutrients, Root System Architectures, Bacterial Communities, and Functional Profiles.” Frontiers in Microbiology 12: 759374. 35082764 10.3389/fmicb.2021.759374 PMC8784561

[emi470370-bib-0025] Hartmann, M. , and J. Six . 2023. “Soil Structure and Microbiome Functions in Agroecosystems.” Nature Reviews Earth & Environment 4, no. 1: 4–18.

[emi470370-bib-0026] Jia, W. , P. Huang , K. Zhu , et al. 2024. “Zonation of Bulk and Rhizosphere Soil Bacterial Communities and Their Covariation Patterns Along the Elevation Gradient in Riparian Zones of Three Gorges Reservoir, China.” Environmental Research 249: 118383. 38331152 10.1016/j.envres.2024.118383

[emi470370-bib-0027] Joseph, P. O. , F. O. Ojomah , and J. B. Abioye . 2025. “Poultry Manure‐Induced Influence on Soil Properties of Coarse‐Textured Tropical Soil.” Journal of Wastes and Biomass Management (JWBM) 7, no. 1: 1–5.

[emi470370-bib-0028] Klein, H. S. , and F. V. Luna . 2021. “The Growth of the Soybean Frontier in South America: The Case of Brazil and Argentina.” Revista de Historia Economica‐Journal of Iberian and Latin American Economic History 39, no. 3: 427–468.

[emi470370-bib-0029] Liu, X. , X. Rong , P. Jiang , et al. 2024. “Biodiversity and Core Microbiota of Key‐Stone Ecological Clusters Regulate Compost Maturity During Cow‐Dung‐Driven Composting.” Environmental Research 245: 118034. 38147920 10.1016/j.envres.2023.118034

[emi470370-bib-0030] Matchado, M. S. , M. Rühlemann , S. Reitmeier , et al. 2024. “On the Limits of 16S rRNA Gene‐Based Metagenome Prediction and Functional Profiling.” Microbial Genomics 10, no. 2: 001203. 38421266 10.1099/mgen.0.001203 PMC10926695

[emi470370-bib-0031] Mishra, A. , L. Singh , and D. Singh . 2023. “Unboxing the Black Box—One Step Forward to Understand the Soil Microbiome: A Systematic Review.” Microbial Ecology 85, no. 2: 669–683. 35112151 10.1007/s00248-022-01962-5 PMC9957845

[emi470370-bib-0032] Modgil, R. , B. Tanwar , A. Goyal , and V. Kumar . 2021. “Soybean ( *Glycine max* ).” In Oilseeds: Health Attributes and Food Applications, 1–46. Springer Singapore.

[emi470370-bib-0033] Nwachukwu, B. C. , A. S. Ayangbenro , and O. O. Babalola . 2023. “Structural Diversity of Bacterial Communities in Two Divergent Sunflower Rhizosphere Soils.” Annals of Microbiology 73, no. 1: 9.

[emi470370-bib-0034] Ogumba, P. , B. Okorie , P. Eleke , et al. 2024. “Prospects of Enhancing Cattle‐Dung Manure's Effectiveness by Partial Substitution With Poultry Droppings‐Compost Mix for Slash‐and‐Burn Managed Tropical Soils.” West Africa Journal of Applied Ecology 32, no. 2: 10–22.

[emi470370-bib-0035] Omotayo, O. P. , O. N. Igiehon , and O. O. Babalola . 2021. “Metagenomic Study of the Community Structure and Functional Potentials in Maize Rhizosphere Microbiome: Elucidation of Mechanisms Behind the Improvement in Plants Under Normal and Stress Conditions.” Sustainability 13, no. 14: 8079.

[emi470370-bib-0036] Rastogi, M. , S. Verma , S. Kumar , et al. 2023. “Soil Health and Sustainability in the Age of Organic Amendments: A Review.” International journal of environment and climate change 13, no. 10: 2088–2102.

[emi470370-bib-0037] Sauma‐Sánchez, T. , J. Alcorta , J. Tamayo‐Leiva , et al. 2024. “Functional Redundancy Buffers the Effect of Poly‐Extreme Environmental Conditions on Southern African Dryland Soil Microbial Communities.” FEMS Microbiology Ecology 100, no. 12: fiae157. 39568064 10.1093/femsec/fiae157 PMC11636270

[emi470370-bib-0038] Sedibe, M. M. , A. M. Mofokeng , and D. R. Masvodza . 2023. “Soybean Production, Constraints, and Future Prospects in Poorer Countries. A Review.” In Production and Utilization of Legumes ‐ Progress and Prospects, edited by M. Hasanuzzaman , 1–15. *IntechOpen* .

[emi470370-bib-0039] Semba, R. D. , R. Ramsing , N. Rahman , K. Kraemer , and M. W. Bloem . 2021. “Legumes as a Sustainable Source of Protein in Human Diets.” Global Food Security 28: 100520.

[emi470370-bib-0040] Singh, Y. , S. Arora , V. Mishra , and A. Bhardwaj . 2022. “Regaining the Agricultural Potential of Sodic Soils and Improved Smallholder Food Security Through Integration of Gypsum, Pressmud and Salt Tolerant Varieties.” Agroecology and Sustainable Food Systems 46, no. 3: 410–431.

[emi470370-bib-0041] Sohn, S.‐I. , J.‐H. Ahn , S. Pandian , et al. 2021. “Dynamics of Bacterial Community Structure in the Rhizosphere and Root Nodule of Soybean: Impacts of Growth Stages and Varieties.” International Journal of Molecular Sciences 22, no. 11: 5577. 34070397 10.3390/ijms22115577 PMC8197538

[emi470370-bib-0042] Thakur, S. , A. K. Pandey , K. Verma , A. Shrivastava , and N. Singh . 2024. “Plant‐Based Protein as an Alternative to Animal Proteins: A Review of Sources, Extraction Methods and Applications.” International Journal of Food Science & Technology 59, no. 1: 488–497.

