# Supplementary material for: Bacterial Diversity and Functional Dynamics in the Soybean ( Glycine max L.) Rhizosphere Under Different Organic Fertilisation
Source: Environ Microbiol Rep. 2026 May 31;18(3):e70370. doi: 10.1111/1758-2229.70370 (PMC13239308; doi:10.1111/1758-2229.70370)
Supplement: Supplementary file 1 — Figure S1: Relative abundance of top Pfam families across the treatments. Figure S2: Relative abundance of top TIGRFAM families across the treatments. Figure S3: Unweighted pair‐group method with arithmetic mean (UPGMA). Figure S4a: MetagenomeSeq analysis between groups (Cattle dung‐Poultry manure). Figure S4b: Metagenome Seq analysis between groups (Control‐Bulk). [file EMI4-18-e70370-s001.doc]

**Supplementary materials**

Supplementary Figure 1: Relative Abundance of Top Pfam Families Across the Treatments

Supplementary Figure 2: Relative Abundance of Top TIGRFAM Families Across the Treatments

Supplementary Figure 3: Unweighted Pair-group Method with Arithmetic Mean (UPGMA)

Supplementary Figure 4a: MetagenomeSeq analysis between groups (Cattle dung - Poultry manure)

Supplementary Figure 4b: Metagenome Seq analysis between groups (Control- Bulk)


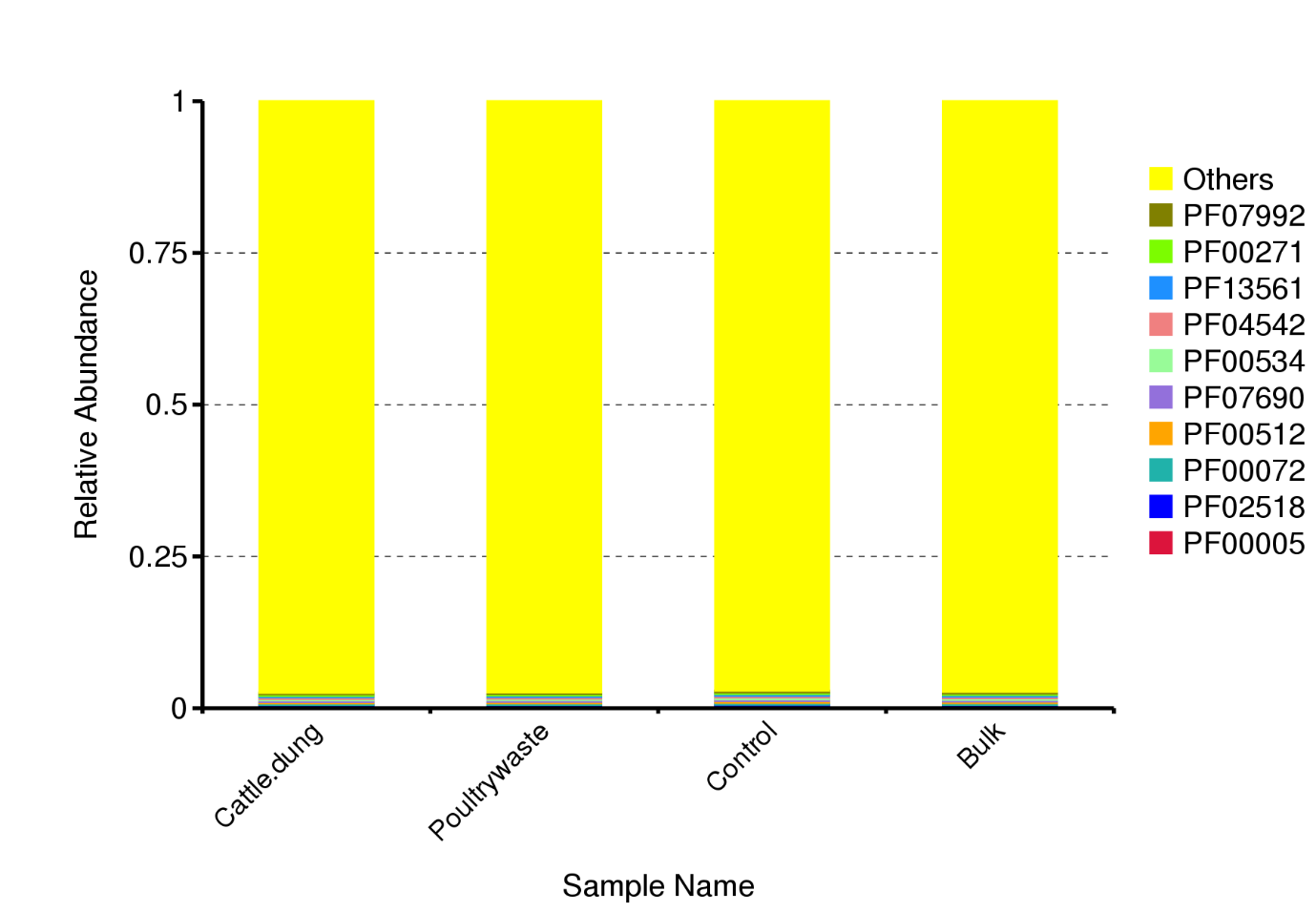


**Supplementary Figure 1**: Relative abundance of top Pfam families across the treatments


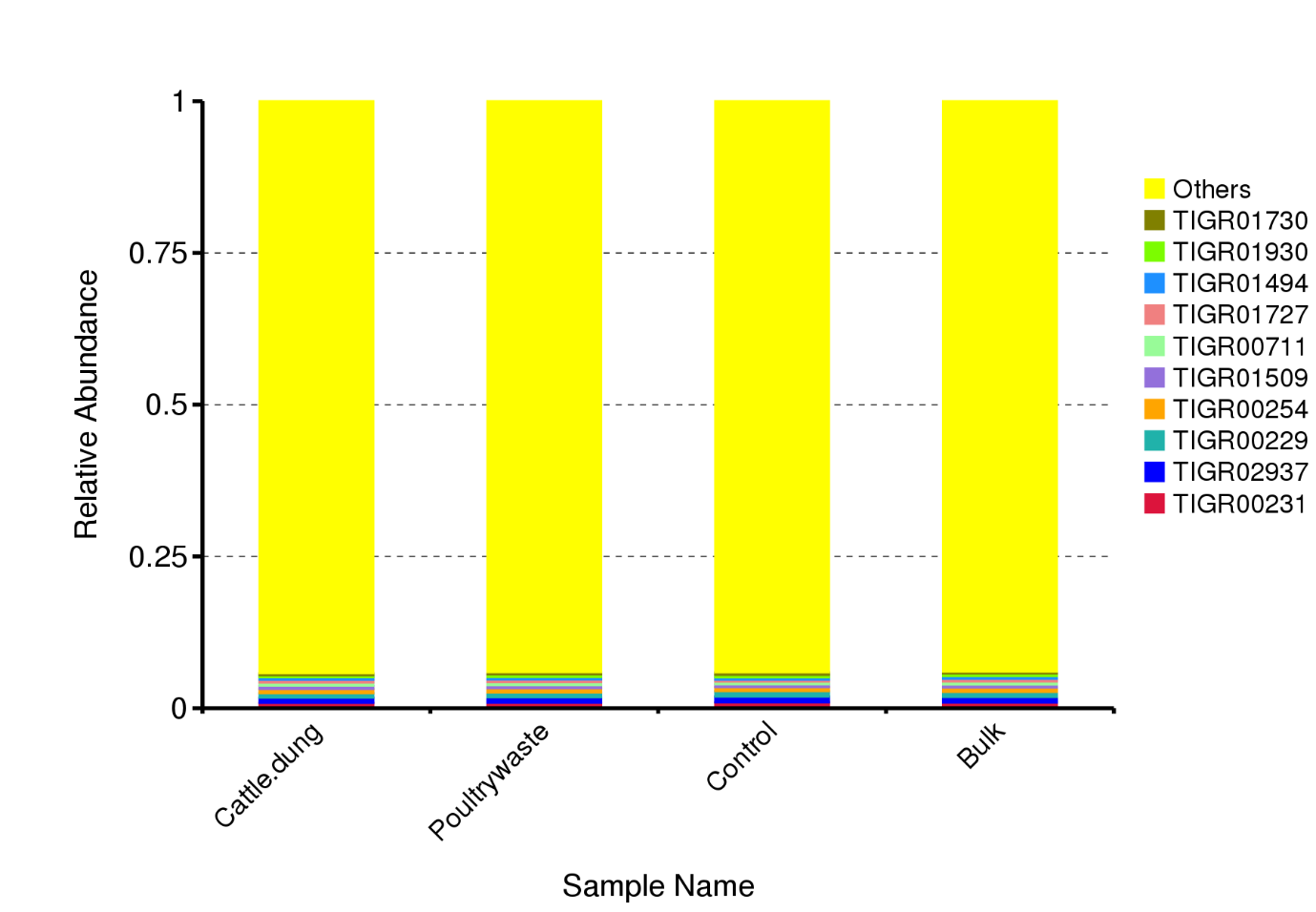


**Supplementary Figure 2**: Relative abundance of top TIGRFAM families across the treatments


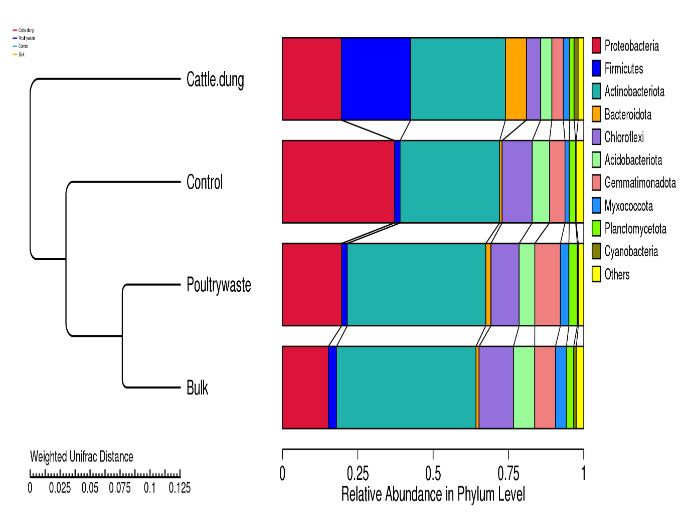


**Supplementary Figure 3**: Unweighted Pair-group method with arithmetic mean (UPGMA)


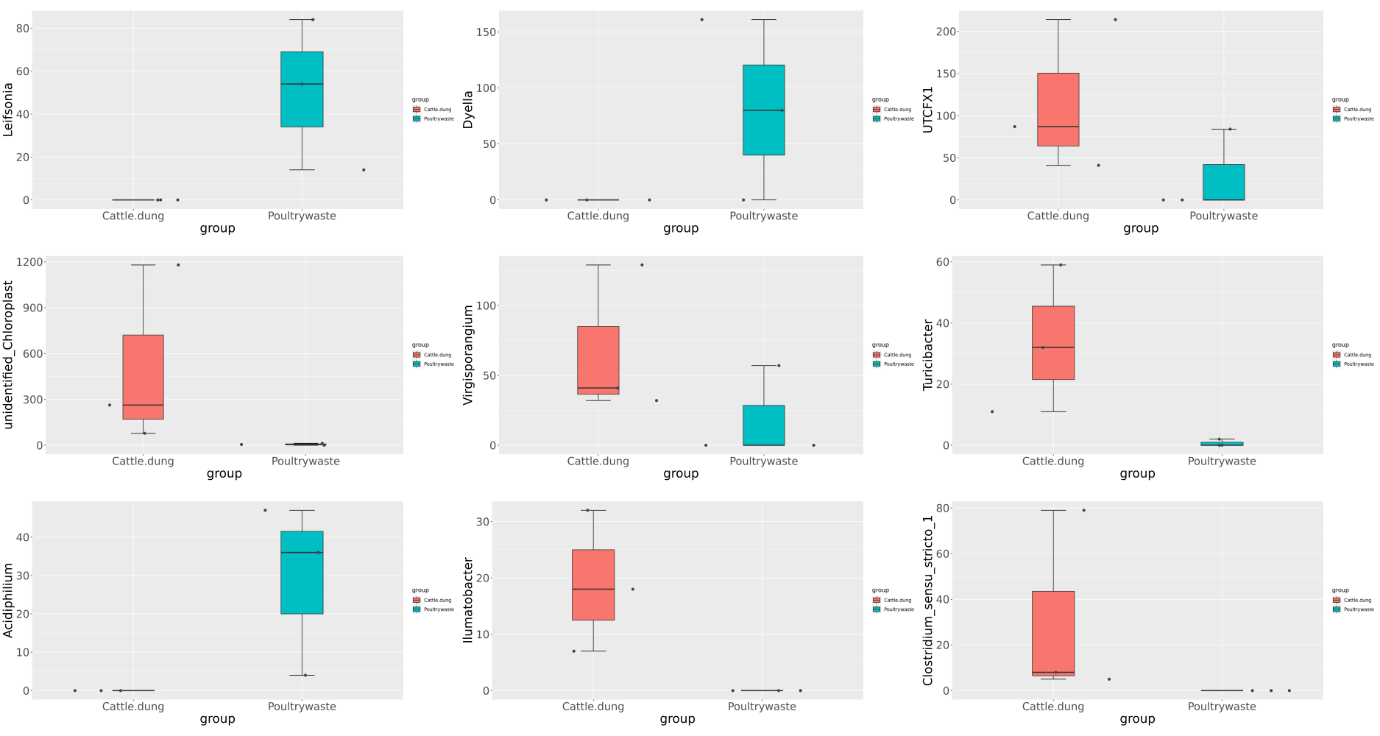


**Supplementary Figure 4a**: MetagenomeSeq analysis between groups (Cattle dung - Poultry manure)


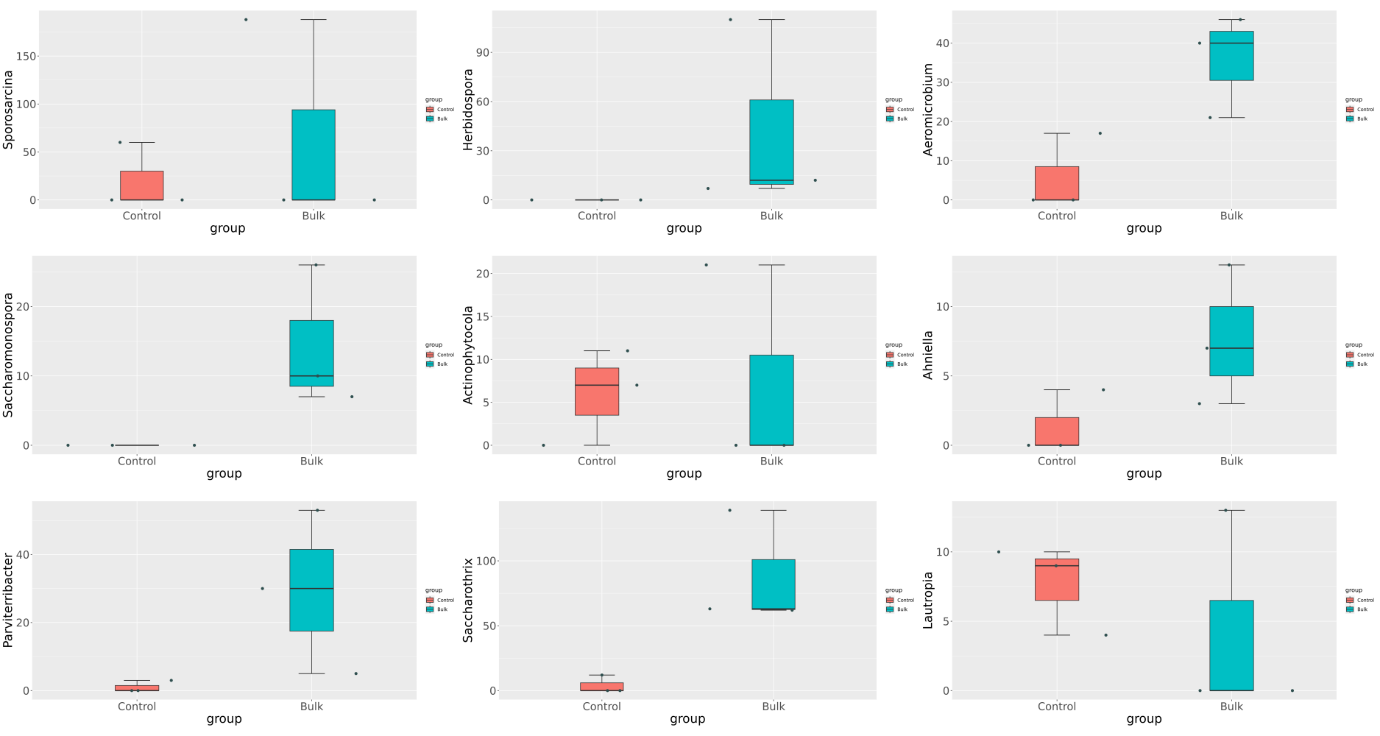


**Supplementary Figure 4b**: Metagenome Seq analysis between groups (Control- Bulk)
